# Metabolic costs of activities of daily living in persons with a lower limb amputation: A systematic review and meta-analysis

**DOI:** 10.1371/journal.pone.0213256

**Published:** 2019-03-20

**Authors:** Loeke van Schaik, Jan H. B. Geertzen, Pieter U. Dijkstra, Rienk Dekker

**Affiliations:** 1 Department of Rehabilitation Medicine, University Medical Center Groningen, University of Groningen, the Netherlands; 2 Department of Oral and Maxillofacial Surgery, University Medical Center Groningen, University of Groningen, the Netherlands; University of Colorado Boulder, UNITED STATES

## Abstract

**Objective:**

To systematically review the literature on the metabolic costs of activities of daily living (ADL) in persons with a lower limb amputation (LLA).

**Data sources:**

A literature search was undertaken in the Pubmed, Embase, CINAHL, CENTRAL, and PsycINFO databases using keywords and synonyms for LLA, metabolic costs, and ADL. The last search was performed on November 29^th^, 2017.

**Study selection:**

Studies were included if they met the following 2 criteria: participants were adults with a (unilateral or bilateral) LLA and metabolic costs were measured while participants performed a physical activity or ADL.

**Data extraction and synthesis:**

Data of 1,912 participants from 61 studies were included in the systematic review and meta-analysis. The studies used different terms to describe metabolic costs. Participants were recruited in different settings, relatively healthy, with few comorbidities. Limited data were available on metabolic costs of other activities than walking with a prosthesis. A linear mixed model analysis was performed based on the means reported, with study as unit of analysis and test results of different groups and measurement conditions as repeated measures within the unit of analysis. Predictors entered in the analysis were e.g. level and reason of amputation, age, weight, and height. During walking, oxygen consumption (ml O_2_/kg/min) and heart rate (beats/min) increased with a higher walking speed and a more proximal amputation. Additionally, oxygen consumption was determined by the interaction terms walking speed x amputation level and walking speed squared. Heart rate was determined by the interaction term walking speed squared.

**Conclusion:**

During walking, oxygen consumption (ml O_2_/kg/min) and heart rate (beats/min) increased with a higher walking speed and a more proximal amputation. Data on metabolic costs of other activities were limited. The poor quality of the studies and the relatively healthy participants limited generalizability of the results of the meta-analysis.

## Introduction

In rehabilitation medicine, the main goals for persons with a lower limb amputation (LLA) are walking with a prosthesis and regaining functional capacity with regard to activities of daily living (ADL) [1–3]. To be able to achieve these goals, certain levels of physical and aerobic capacity are required [4,5].

In the Netherlands, more than 90% of LLAs are due to vascular disease and/or diabetes mellitus (DM) [6,7]. In the UK and USA, 75% and 87% of LLAs are due to vascular disease, respectively [8]. Persons with a LLA who have (peripheral) vascular disease (PVD) are mostly elderly and have comorbidities resulting from atherosclerosis, which limits their physical and aerobic capacity [9–11]. In general, aerobic capacity decreases with age, and studies have shown that elderly participants with a LLA have a lower aerobic capacity than controls [12,13]. The VO_2_max test is the criterion measure of aerobic capacity, and it is a valid predictor of cardiorespiratory capacity [14]. To date, however, few studies have measured VO_2_max in persons with a LLA. Furthermore, these studies used different test protocols [10,15,16]. They reported lower levels of VO_2_max in participants with a LLA compared with controls. These differences are probably due to deconditioning or comorbidities.

A previous systematic review looked at the influence of physical capacity on regaining the ability to walk (with a prosthesis) [17]. No sufficient evidence was found for aerobic and anaerobic capacity as potential predictor for walking ability. Another systematic review investigated factors that predict walking ability after a LLA [18]. One of these factors was fitness of the participants. The main finding of this review was the substantial heterogeneity in testing methods and outcome measures of the included studies, which hampered a meta-analysis. Both systematic reviews did not primarily aim to analyse metabolic costs in participants with a LLA, and both only reviewed walking as activity.

One study reported the relative aerobic load of walking, measured as a percentage of the VO_2_peak [19]. The relative aerobic load of walking was higher in participants with a LLA than in controls. Another study reported that participants with a LLA who had higher levels of physical fitness (defined as the maximum oxygen uptake during exercise as a proportion of predicted maximum oxygen uptake) were more likely to walk with a prosthesis than participants with a LLA with lower levels of physical fitness [10].

For the general population, the metabolic costs of ADL are well reported in the compendium of physical activities, the so called Metabolic Equivalent of Task (MET)-values [20]. However, these reference values seem not to apply for persons with a disability. For example, in persons after stroke, the required metabolic costs for certain activities are higher compared to the MET-values [21]. For persons with LLA knowledge about the required metabolic costs for ADL, in combination with maximum aerobic capacity, is lacking. However, this knowledge is relevant for optimizing training programs and setting functional goals for rehabilitation.

The specific aims of this systematic review and meta-analysis are 1) to analyse what is known about the metabolic costs of different ADL in participants with a LLA; 2) to explore which methods and outcome measures are used to evaluate these metabolic costs; and 3) to determine whether metabolic costs are influenced by level of amputation, reason for amputation, and walking speed.

## Methods

The Preferred Reporting Items for Systematic Reviews and Meta-Analyses (PRISMA) was used for conducting this systematic review and meta-analysis [22].

### Protocol

The protocol for this systematic review and meta-analysis was registrated at PROSPERO (https://www.crd.york.ac.uk/PROSPERO/#index.php, CRD42016050990).

### Study identification and selection

A literature search was performed in the Pubmed, Embase, CINAHL, CENTRAL, and PsycINFO databases. No language or date restrictions were applied. The last search was performed on November 29^th^, 2017. Different search terms for LLA were used, combined with various search terms for metabolic costs and physical activities and/or ADL (S1 File). Two reviewers (LvS, RD) independently assessed titles and abstracts. In case of disagreement between reviewers, the record was included for full text analysis. Full text assessment was performed by the aforementioned reviewers, who used the same criteria for inclusion and exclusion. Any further disagreements were resolved by consensus through discussion. Cohen’s kappa was calculated for the title and abstract assessment and the full text assessment of the selection process. The reference lists of studies included in the systematic review were checked for other relevant studies, which were subsequently assessed following protocol. When after full text assessment more than 50 studies resulted, studies with a total study population < 10 were excluded because the outcomes lacked precision.

### Inclusion and exclusion criteria

Inclusion criteria were as follows: participants were adults with a (unilateral or bilateral) LLA and metabolic costs were measured while participants performed a physical activity or ADL. Editorials, (expert) opinions, comments, reviews, and off- topic studies were excluded. Studies on toe or midfoot amputations, amputations other than a lower extremity, or endoprostheses were also excluded, as were studies with children or animals and model studies.

### Quality assessment

Quality assessment was independently performed by two reviewers (LvS, RD) using the Agency for Healthcare Research and Quality (ARHQ) methodology checklist [23]. Additionally, it was assessed whether equipment for measuring oxygen consumption was validated and whether walking speed during testing was reported.

### Statistical analyses

Statistical analyses were performed using IBM SPSS Statistics 23. Descriptive statistics (mean and standard deviation) were calculated for study variables. Initially, effect sizes were calculated based on study data. For oxygen consumption one study, however, reported uncommonly small standard deviations relative to other studies, resulting in extremely large effect sizes [24]. The authors were contacted to verify whether the standard deviations were truly standard deviations or perhaps standard errors of the mean. The authors did not respond. Additionally, 7 studies did not reported standard deviations [25–31]. For heart rate, 5 studies did not report standard deviations [25,29–32]. Therefore a linear mixed model analysis was performed (maximum likelihood method and AR1 covariance structure) on the means reported. The study was used as a unit of analysis and the study results of different groups and measurement conditions were used as repeated measures within the unit of analysis (multilevel structure). Forest plots were not made because effect sizes were not used in the analysis.

The outcome oxygen consumption (expressed as ml O_2_/kg/min) was analysed using the following potential predictors: level of amputation, reason for amputation, age, weight, height, and walking speed. Only studies that reported oxygen consumption (ml O_2_/kg/min) were selected. When possible, outcomes were converted to ml/kg/min. If this was not possible based on the reported data within the study, the study was excluded for the analysis. Potential predictors were entered into the linear mixed model analysis. If the model fit (-2LL statistic) increased significantly, the predictors remained in the model. Cause of amputation was categorized in trauma and vascular, level of amputation as below knee and above knee. Studies with mixed groups (when it was not possible to analyse subgroups based on level and reason of amputation) were excluded from the analysis.

In a few studies [16,33–35] (n = 4) a treadmill with an incline was used. Data regarding treadmills with an incline were not included in the meta-analysis because of the small number of studies. Some studies did not specify whether the walking surface was level. The inclusion or exclusion of those studies in the meta-analysis did not affect the results. Therefore, studies that did not specify the treadmill incline were included, and it was assumed the walking surface was level. Interaction terms of the predictors significantly associated with oxygen consumption were explored. A similar analysis was performed for heart rate as an outcome measure.

## Results

### Study inclusion

**Fig 1:**
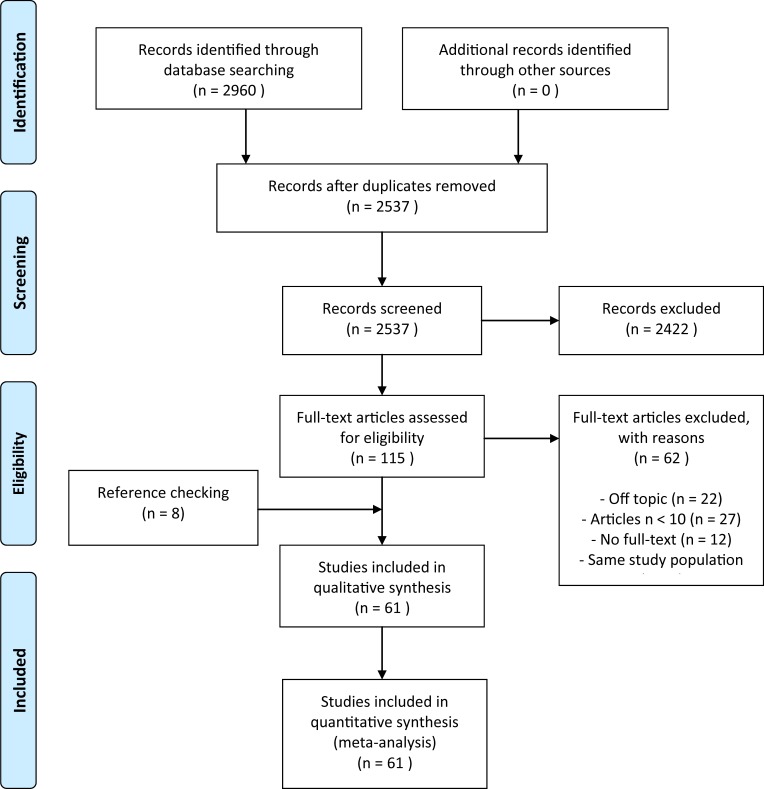
Flowchart showing the inclusion process.

A total of 2,960 potentially suitable records were found in the database search. After removing duplicates, 2,537 records remained (Fig 1). After title and abstract assessment, 2,422 records were excluded (agreement 98%, Cohen’s kappa: 0.71), leaving 115 records for full text assessment. Of 12 records, no full text was available (yet). After full-text analysis, 22 studies were excluded because they were off topic (agreement was 96%, Cohen’s kappa: 0.88). A further 8 studies were identified from the reference lists and were assessed for inclusion, resulting in a total of 89 studies. Given the fact that more than 50 studies were identified, studies with a sample size less of than 10 participants were excluded (n = 27). Two studies were found [36,37] that partially included the same study population (in one study persons with a Syme amputation were included and in the other study they were excluded). The former study was included in this study [36]. In total 61 studies were included. None of the included studies were written in another language than English or Dutch.

### Participant characteristics

The 61 included studies (n = 1,912) were heterogeneous. The mean (SD) number of participants was 13.5 (13.2). The groups ranged from 10 to 101, and subgroups ranged from 3 to 44. There were different reasons for amputation and/or levels of amputation. Time since amputation ranged from 68 days to 27 years (Table 1). The majority of participants were male (68%, n = 1,299), and for 17% (n = 324) of the participants gender was not specified. The majority of studies (n = 57) included (subgroups of) participants with a transtibial amputation or a transfemoral amputation. A few studies [38–40] included participants with other levels of amputation. In 12 studies data of participants with different levels of amputation were pooled into a single group. The reason for amputation was not described in 8 studies. Eighteen studies included mixed groups with regard to reason for amputation, and in 8 studies the reason for amputation was described as ‘other than vascular.’ In 19 studies the use of walking aids was described (or it was stated explicitly that no aids were used). Comorbidities were reported in 8 studies [10,11,40–45].

**Table 1:** Participant characteristics of the included studies.

Authors, year	n (% men) vs. Control (% men)	Age ±SD (range)	Level amputation (% n)	Reason amputation (%)				Years since amputation ±SD (range) *months
				vasc	trauma	onco	other	
Ganguli, 1973[29]	10 (-)	29.9±11	TT (100)	-	-	-	-	-
	C 16 (100)	28.4±7.1	n.a.					
James, 1973[33]	37 (100)	42.8±12.8	TF (100)	-	-	-	-	18 (2–48)
	C 26 (100)	39.6±14.0	n.a.					
Ganguli, 1974[48]	6 (-)	26.2±10.2	TT (100)	-	-	-	-	6–12*^†^
	C 6 (100)	34.5±6.2	n.a.					
Ganguli, 1975[30]	10 (100)	27.3±7.1	LLA^‡^	-	-	-	-	-
	10 (100)	29.9±1	TT (100)	-	-	-	-	-
	C 16 (100)	28.4±7.1	n.a.					
Waters, 1976[4]	13 (-)	60	TF (100)	100				1.2
	13 (-)	63	TT (100)	100				1.4
	15 (-)	57	Syme (100)	100				1.1
	15 (-)	31	TF (100)		100			10.0
	14 (-)	29	TT (100)		100			14 (-)
	C 50 (-)	-	n.a.					
Huang, 1979[49]	6 (83)	30.4± 8.7	TF (100)	*-*	*-*	*-*	*-*	*-*
	6 (50)	38.2±12.9	TT (100)	*-*	*-*	*-*	*-*	*-*
	4 (50)	33.5±12.1	TF bil (100)	*-*	*-*	*-*	*-*	*-*
	C 25 (20)	(19–43)	n.a.					
Pagliarulo, 1979[50]	15 (80)	28.9±11.2	TT (100)				100^§^	> 1^†^
DuBow, 1983[32]	6 (83)	61±11.3	TT bil (100)	100				1.9±1.1^†^
	C 8 (75)	55±7	n.a.					
Nowroozi, 1983[38]	8 (63)	36.75±17.75	HD (100)			100		-
	10 (70)	40±13.2	HP (100)					-
	C 11 (45)	30.4±10.9	n.a.					
Isakov, 1985[11]	14 (93)	60.5 (50–75)	TF (100)	100				1*
	3 (100)	35.3 (26–48)	TF (100)		100			> 5
Pinzur, 1992[42]	25 (-)	57.8	TF (20), KD (20), TT (20), Syme (20), midfoot (20)	100				> 6*^†^
	C 5 (-)	54.5	n.a.					
Gailey, 1993[51]	10 (100)	37.2±11.0	TF (100)				100^§^	13.6
	10 (100)	34.6±9.8	TF (100)					15.4
	C 10 (-)	33.2±9.6	n.a.					
Jaegers, 1993[52]	11 (100)	-	TF (100)				100^||^	-
	C 6 (100)	-	n.a.					
Boonstra, 1994[53]	29 (83)	41±13	TF (100)	3.5	59	34	3.5	19±13
Gailey, 1994[25]	39 (100)	47±16	TT (100)				100^§^	> 6*
	C 21 (100)	31±6	n.a.					
Torburn, 1995[43]	10 (100)	50.6±15.6	TT (100)		100			-
	7 (100)	62.0±8.3		100				-
Hoffman, 1997[54]	5 (80)	22±3	TF bil (100)		60		40	12.4 (2–24)
	C 5 (80)	22±6	n.a.					
Chin, 2002[10]	8 (-)	72.2±2.1	TF (100)	100				-
	9 (-)	63.2±2.1	TF (100)	100				-
Schmalz, 2002[55]	7 (100)	49±17	TT (100)		100			23±19
	8 (100)	22±17						18±17
	6 (100)	33±6	TF (100)					13±6
	6 (100)	37±9						13±9
Bussmann, 2004[56]	10 (90)	64.6±9.6	TF (20), KD (20), TT (60)	-	-	-	-	68 days (39–131 days)
	C 10 (-)	61.3±11.4	n.a.					
Datta,2005[46]^¶^	10 (70)	38 (23–46)	TF (100)		80	20		≥5
Chin, 2006[57]	4 (100)	24.0±7.6	TF (100)		75	25^#^		-
	C 14 (71)	25.2±4.0	n.a.					
Chin, 2006[39]	34 (71)	67.0±5.6	HD (15), TF (85)	29			71^§^	-
	15 (67)	67.1±5.7	HD (7), TF (93)	60			40^§^	-
Paysant, 2006[24]	10 (100)	39.2 (21–65)	TT (100)		100			17.4 (2–38)
	C 20 (100)	39.7	n.a.					
Hagberg, 2007[58]	41 (73)	49±11.5	TF (100)		71	24	5	27±14.5
	C 22 (73)	49±8.3	n.a.					
Seymour, 2007[59]	13 (85)	46±13	TF (92), KD (8)				100^§^	16±15*^†^
Bussmann, 2008[60]	9 (100)	55.4 (21–73)	TT (100)		100			15.6 (3–61)
	C 9 (100)	55.9 (21–76)	n.a.					
Genin, 2008[61]	10 (100)	34.7±5.1	TF (53)		100			11.2±4.2
	9 (100)	35.3±7.3	TT (47)					
	C 13 (77)	27.8±5.2	n.a.					
Kaufman, 2008[62]	15 (80)	42.9±9	TF (100)	7	47	40	7	20±10
Traballesi, 2008[44]	16 (69)	61±11	TF (67)	100				-
	8 (75)	56±17	TT (33)					-
Wright, 2008[63]	10 (100)	40.5±11.9	TT bil (20), TF bil (30), KD bil (10), TT/TF (40)		80		20	> 22 (2–48)
Hamamura, 2009[40]	44 (64)	66.7±5.1	HD (23), TF (77)	27			73^§^	-
	20 (60)	68.7±5.6	HD (5), TF (95)	55			45^§^	
Houdijk, 2009[64]	11 (-)	46±9	TT (100)	27	73			>1^†^
	C 11 (-)	47±11	n.a.					
Tekin, 2009[34]	10 (100)	27.7±5.3	TT (100)		100			50.3±54.2*
	C 9 (100)	28.4±4.2	n.a.					66.1±49.6*
Goktepe, 2010[35]	64 (100)	29.1±4.5	TF (15), TT (50), partial foot (35)		100			62.6±50.9*
Andrysek, 2011[65]	19 (86)	33.4	TF (93), KD (7)	7	57	21	14	13.2
Hagberg, 2011[66]	28 (71)	49±14.3	HD (7), TF (46), KD (18), TT (29)		61	29	11	18±17^†^
	C 31 (65)	47±10.2	n.a.					
Kark, 2011[67]	6 (67)	65±18	TF (100)	17	83			median 22.5 (IQR 40.8)
	10 (80)	62±20.8	TT (100)	20	80			median 8.0 (IQR 26.8)
	C 28 (43)	59.0±13.0	n.a.					
Mohanty, 2012[68]	30 (87)	34.1±4.4	TT (100)		100			>1*^†^
Schnall, 2012[26]	12 (100)	26.9±5.5	TT (100)		100			≥ 6*
	C 12 (100)	20.9±2.8	n.a.					
Sokhangoei, 2013[31]	24 (100)	33 (20–40)	TT (100)		100			13.7±6.8
	C 24 (100)	29.3	n.a.					
Wezenberg, 2013[19]	10 (80)	66.3±5.9	TF (30), TT (70)	100				3.8±3.6
	26 (69)	60.7±5.6	TF (38), TT (62)		100			36.2±20.7
	C 21 (67)	60.8±5.9	n.a.					
Bell, 2014[69]	26 (-)	32±6.1	TF (100)		100			≥2
Erjavec, 2014[41]	101 (63)	69.4 (53–84)	TF (100)	100				-
Esposito, 2014[70]	13 (100)	28.9±5.3	TT (100)		100			6.6±6.2*
	C 13 (100)	26.5±6.0	n.a.					
Gjovaag, 2014[16]	12 (50)	42.8±13.5	TF (100)				100^§^	≥2
	C 12 (50)	43.0±11.7	n.a.					
Rowe, 2014[71]	17 (88)	52.2±12.9	TT (100)	59	12		29	8.3±7.6
Vllasolli, 2014[37]	22 (91)	40.6±12.5	TF (100)		91		9	17.1±10.5
	61 (85)	39.7±13.1	TT (100)		95		5	14.5±7.5
	6 (83)	36.2±6.2	Syme (100)		100			11.3±2.4
Delussu, 2016[72]	20 (85)	66.6±6.7	TT (100)	65	30	5		0.5^†^
Esposito, 2016[73]	6 (83)	29±6	TT (100)		100			2 *^†^
	C 6 (83)	23±5	n.a.					
Guirao, 2016[45]	10 (60)	50.3±16.1	TF (100)	40	40	20		8.1
Starholm, 2016[74]	8 (50)	37.0±10.9	TF (100)				100^§^	≥2
	C 8 (50)	39.0±12.3	n.a.		100			27±22*
Andrysek, 2017[75]	10 (60)	20.9±3.1	TF (100)		40		60	6.8±4.5
Esposito, 2017[76]	14 (-)	27±5	TF (100)		100			23±11*
	C 14 (-)	26±6	n.a.					
Gardinier, 2017[77]	10 (100)	46.5 (20–60)	TT (100)	-	-	-	-	>6*
	C 10 (100)	48.4 (20–63)	n.a.					
Gjovaag, 2017[28]	8 (50)	37.0±10.9	TF (100)				100^§^	15.9±13.9^†^
	C 8 (50)	39.0±12.3	n.a.					
Jarvis, 2017[27]	10 (100)	28±4	TT (100)		100			39±27*
	10 (100)	29±3	TF (100)		100			35±7*
	10 (100)	29±4	TF bil (100)					
	C 10 (100)	30±6	n.a.					
Lacraz, 2017[78]	15 (75)	46.3±12.7	TT (100)		100			17.6±15.2
Ladlow, 2017[79]	10 (100)	32±5	TT (60), KD (20), TF (20)		100			24±15*
	10 (100)	29±4	TT bil (10), KD bil (20), TF bil (30), TT/TF (20), KD /TF (20)		100			39±14*
	C 10 (100)	32±6	n.a.					
Mutlu, 2017[80]	13 (-)	44.0±15.9	TF (-), TT(-), Syme (-)	-	-	-	-	15.6±14.2^†^
Weinert, 2017[81]	8 (-)	38±3	TT (100)		100			≥0.5^†^
	9 (-)	28±4	TF (100)		100			≥0.5^†^
	10 (-)	29±4	TF bil (100)		100			≥0.5^†^
	C 10 (-)	29±4	n.a.					

N number participants; C controls;—not reported; vasc vascular; onco oncology; TT transtibial amputation; n.a. not applicable; KD knee disarticulation; TF transfemoral amputation; bil bilateral; HP hemipelvectomie; HD hipdisarticulation; IQR interquartile range

* months since amputation reported with 1 decimal, no decimal if not reported in the studies

^†^ prosthetic years

^‡^ not reported in text or table what level of amputation

^§^ reported as nonvascular

^||^ trauma or osteosarcoma, no percentages/numbers reported

^¶^ data reported in other study

^#^ data in text and table differ from each other within the study, data from the table were used

One study[46] referred to a previous study for the participant characteristics[47].

### Study quality

Agreement for the quality assessment was 93% (Cohen’s kappa: 0.89). The mean quality score was 6.6, ranging from 3 to 10 points out of 14 possible points (S1 Table). In 43 of the 61 studies inclusion criteria were reported. Only 22 studies reported exclusion criteria. In 8 studies the timeframe of recruitment was reported. The majority of studies (n = 56) reported walking speed(s). Most studies (n = 56) used validated measurement equipment. In a few studies (n = 5) the type of validation was not explicitly specified.

### Study characteristics

All studies had an observational design. Three studies [32,42,68] tested the same measurement conditions repeatedly. Almost all studies tested walking as an activity, but there was a great variety in the applied test protocols, test surroundings, and tested walking speeds between studies (Table 2). Furthermore, different terms for metabolic costs were used, as well as different outcome measures. Oxygen consumption (ml O_2_/kg/min) and heart rate (beats/min) were the most frequently reported outcome measures of metabolic costs in 39 and 36 studies, respectively (Table 2, S2 Table). Therefore, these 2 outcome measures were used in the meta-analysis.

**Table 2:** Study characteristics of the included studies.

Author, year	Activity	Surrounding	Metabolic cost outcome measures	Walking speed (km/h)*	Oxygen consumption mean(SD) for walking	Heart rate mean(SD) for walking
Ganguli, 1973	Sitting, standing up, stand erect, walking, stair ascending, stepping	Indoor	Oxygen consumption (l/min), energy expenditure † (cal/min/kg), peak HR†	3	-	-
James, 1973	Walking level and 5° inclination	Treadmill	HR, oxygen uptake (l/min/kg), blood lactate	1.5, 2.7 and 3.9	-	TF 95(2), 104(2), 118(2) 5° inclination 103(2), 119(2), 148(2) Controls: 87(2), 93(2), 95(2) 5° inclination 92(2), 103(2), 116(2)
Ganguli, 1974	Walking	Indoor	Energy expenditure (kcal/kg/km and kcal/km), peak HR‡	3, 4 and 5	-	TT 102(18), 116(21), 113(23) Controls 100(10), 105(9), 112(9)
Ganguli, 1975	Sitting, standing up, stand erect, walking, stair ascending, stepping	Indoor	Energy expenditure (kcal/min) and peak HR	3	-	TT 114(-) Controls 94(-)
Waters, 1976	Walking with prosthesis and with crutches (without prosthesis)	-	oxygen uptake (ml/kg/min), net oxygen cost (ml/kg/m), relative energy cost (%), HR, RQ	SSWS	TF vasc 12.6(2.9), TT vasc 11.7(1.6), Syme vasc 11.5(1.5), TF trauma 12.9(3.4), TT trauma 15.5(2.9)	TF vasc 126(17), TT vasc 105(17), Syme vasc 108(13), TF trauma 111(12), TT trauma 106(11)
Huang, 1979	Walking	Indoor and outdoor	Energy cost (cal/ft/kg), oxygen consumption (ml/ft/kg)	SSWS	-	-
Pagliarulo, 1979	Walking with prosthesis and with crutches (without prosthesis)	Outdoor	HR, oxygen consumption, energy cost (ml/kg/min en ml/kg/m), RR, BP	SSWS, slow and fast	With prosthesis: 15.5(2.8), without prosthesis 22.3(4)	With prosthesis 106(10), without prosthesis 135(22)
DuBow, 1983	Walking and wheelchair	Indoor	Oxygen consumption (ml/min/kg), HR, %PMHR	SSWS and wheel ergometer	Bil.TT 7.8(2.2),controls 6.9(1.7)	Bil.TT 116(-), controls 92(-)
Nowroozi, 1983	Walking	-	Oxygen consumption (ml/min/kg), HR	SSWS, slow and fast	HD SSWS 11.1(1.7), SSWS slow 9.3(2.1), SSWS fast 14.5(0.9) HP SSWS 11.5(3.5), SSWS slow 8.8(3.7), SSWS fast 13.7(4.9) Controls SSWS 9.8(1.8)	HD SSWS 99 (-), SSWS slow 105(-), SSWS fast 123(-) HP SSWS 97(-), SSWS slow 92(-), SSWS fast 115(-)
Isakov, 1985	Walking	-	Increase in HR and oxygen consumption (ml/min)	SSWS	-	-
Pinzur, 1992	Walking	Treadmill	Oxygen consumption (ml/min/kg)	Rest, normal and maximal	-^#^	-
Gailey, 1993	Walking	Indoor	Oxygen uptake (-), HR	2 and 4	TF CAT CAM 10.4(1.3) and 15.1(1.9) TF QUAD 11.7(2.7) and 19.0(5.5) Controls 8.5(1.1) and 11.1(1.9)	TF CAT CAM 101(13) and 116(15) TF QUAD 101(11) and 120(16) Controls 84(9) and 90(9)
Jaegers, 1993	Walking	Treadmill	Oxygen uptake (l/min), HR	SSWS + 6 different speeds	-	
Boonstra, 1994	Walking	Treadmill	Energy expenditure (J/s/kg)	2 and 3	-	
Gailey, 1994	Walking	Indoor	Oxygen uptake (l/min and l/min/kg), HR	SSWS	12.9(-), Controls 10.9(-)	103(-). Controls 87(-)
Torburn, 1995	Walking	-	HR, energy consumption (ml/kg/min), RQ	SSWS	^$^TT trauma SACH 18.4(3.0), Carbon Copi II 18.0(3.6), Seatle light 17.2(3.6), quantum 17.1(2.7), flex-foot 17.8(3.5) TT vasc SACH 13.4(2.8), Carbon Copi II 13.6(1.7), Seatle light 13.7(2.7), quantum 13.1(2.2), flex-foot 12.4(2.3)	-^#^
Hoffman, 1997	Walking	Indoor	Oxygen uptake (l/min), HR	SSWS, 1.2, 2.2 and 3.3	-^#^	-^#^
Chin, 2002	Cycling	Indoor one leg cycling	%VO2max	n.a.	-	-
Schmalz, 2002	Walking	Treadmill	Oxygen rate (ml/min/kg), HR	Different speeds	^$^TT trauma 1S71 13.5(0.9) and 16.1(1.4), 1D10 13.3(0.8) and 15.5(1.5), 1D25 13.6(0.7) and 15.7(1.2), 1C40 13.5(0.9) and 15.7(1.2), flex foot 13.6(1.2) and 15.6(1.2). TF trauma SSWS 3C1 15.1(1.1), C-leg 14.2(1.2). SSWS slow 3C1 12.9(0.9), C-leg 12.1(1.1), SSWS fast 3C1 16.8(1.4), C-leg 16.2(2.1)	-
Bussmann, 2004	Walking	Indoor	HR rest, HR during walking, % HRR	SSWS and fixed-speed test (speed increased every min)	-	-
Datta,2005	Walking	Treadmill	Oxygen cost (ml/kg/m)	Start 2.5, 0.5 increments at 3min interval, up to 5	-^#^	-
Chin, 2006	Walking	-	Oxygen uptake (ml/kg/min) Oxygen cost (ml/kg/m)	1.8, 3.0, 4.2 and 5.4	TF C-leg 11.6(2.6), 15.6(4.3), 20.1(3.6), 26.9(5.2) TF IP 12.4(5.7), 16.3(4). 21(4.3), 28.1(5.4) Controls 8.7(1.9), 10.4(2.5), 13.3(3.1), 17.3(3.2)	-
Chin, 2006	Cycling	-	%VO2max	n.a.	-	-
Paysant, 2006	Walking (asphalt, mown lawn and high grass)	Outdoor	Oxygen uptake (ml/kg/min), oxygen cost (ml/kg/m), HR	-	TT gras flat 15.1(0.2), grass uneven 18.3(0.2), asphalt 14.6(0.2). Controls grass flat 14.1(0.2), grass uneven 15.7(0.1), asphalt 13.4(0.2)	TT gras flat 101(9), grass uneven 115(17), asphalt 101(16). Controls grass flat 103(12), grass uneven 107(14), asphalt 99(10)
Hagberg, 2007	Walking	Indoor	HR, PCI	SSWS	-	111(16) Controls 94(14)
Seymour, 2007	Walking	Treadmill	HR, oxygen consumption (ml/kg/min), oxygen cost (ml/kg/m)	SSWS and fast SSWS	TF/KD SSWS C-leg 12.6(1), NMC 13.5(2) TF/KD SSWS fast C-leg 16.0(2), NMC 17.2(2)	TF/KD SSWS C-leg 102(14), NMC 103(16) TF/KD SSWS fast C-leg 102(16), NMC 104(15)
Bussmann, 2008	Walking	-	HR rest, HR during walking, % HRR	-	-	91(16) Controls 90(16)
Genin, 2008	Walking	Outdoor	Gross cost (J/kg/m), net cost (J/kg/m)	1.1 to 8.3	-	-
Kaufman, 2008	Walking	Treadmill	Objective measurements of energy efficiency (ml/kg/m)	1.6, 3.2 and 3.8	-	-
Traballesi, 2008	Walking	Treadmill and indoor	HR, energy cost (ml/kg/m)	SSWS	TT treadmill 12.3(2.5), floor 13.5(2.4) TF treadmill 13.0(3.5), floor 13.2(3.1)	TT treadmill 106(28), floor 110(27) TF treadmill 108(14), floor 110(13)
Wright, 2008	Walking	Indoor	HR, PCI	SSWS	-	104(16) Controls 85(-)
Hamamura, 2009	One leg cycling test	Indoor	%VO2max	n.a.	-	-
Houdijk, 2009	Walking	Treadmill	Metabolic energy consumption (J/kg/s) and metabolic energy cost (J/kg/m)	SSWS and 4.7	-	-
Tekin, 2009	Walking	Treadmill	EEI (ml/kg/min)	1.5 and 3, 0° en 5° inclination	TT trauma 7.5(1.3), 9.5(2.1), 5° inclination 8.3(1.8) and 10.3(2.4). Controls (salvage) 7.6(1.20), 9.5(2.1), 5° inclination 8.3(1.8), 11.0(1.4)	-
Goktepe, 2010	Walking	Treadmill	oxygen consumption (ml/kg/min), oxygen cost (ml/kg/m)	1.5 and 3, 0° en 5° inclination	TT 7.1(1.7), 9.3(2.4), 5° inclination 7.6(1.8) and 10.9(2.4). TF 7.7(2.1), 10.8(2.2), 5° inclination 8.4(2.0), 11.2(1.9)	-
Andrysek, 2011	Walking	Indoor	HR, PCI	SSWS and fast SSWS	-	-
Hagberg, 2011	Walking	Indoor, test-retest	HR, PCI	SSWS	-	106(15), 108(17) Controls 96(12), 97(12)
Kark, 2011	Walking	-	HR, oxygen consumption (ml/kg/min), oxygen cost (ml/kg/m)	SSWS	-^#^	-
Mohanty, 2012	Walking with prosthesis and walking with axillary crutches without prosthesis	Indoor	Oxygen uptake (ml/min), HR, energy expenditure (kcal/min)	SSWS	-	Walking with prosthesis 82(6), walking with crutches 91(7)
Schnall, 2012	Walking and with 32.7kg load	Treadmill	Oxygen consumption (ml/kg/min)	4.8 and 5.5	TT trauma 22.2(-), 26.4(-) Controls 20.4(-), 23.8(-)	-
Sokhangoei, 2013	Walking	Treadmill	HR, PCI	2, 3 and 4	-	TT 108(-), 113(-), 123(-). Controls 98(-), 101(-), 108(-)
Wezenberg, 2013	Walking Cycling	Treadmill	Peak oxygen consumption (ml/kg/min), oxygen cost (ml/kg/m), energy expenditure walking (ml/kg/min)	SSWS ± 15% en 30% One leg cycling test	Trauma 13.5(2.2) Vasc 12.2(2.5) Controls 13.8(2.1)	-
Bell, 2014	Walking	-	HR, oxygen cost (ml/kg/min)	SSWS 4 and 4.6	17.3(5), 17.3(2.7)	127(18), 124(23)
Erjavec, 2014	Walking and hand wheel ergometer	-	Oxygen uptake (ml/kg/min), HR	6MWT	-	114(-)
Esposito, 2014	Walking	Treadmill	Oxygen consumption (ml/kg/min), HR	SSWS and 5 standardized velocities	TT 9.5(1.1), 10.9(0.9), 12.7(1.1), 15.5(1.6), 19.1(2.3). Controls 9.6(1.0), 10.9(1), 12.8(1.1), 15.5(1.6), 12.8(1.1), 18.9(1.8)	TT 92(11), 97(12), 105(13), 116(15), 130(18). Controls 79(14), 83(14), 88(14), 95(14), 103(15)
Gjovaag, 2014	Walking and running	Treadmill	VO2max (ml/kg/min), HR, RER, Walking economy (ml/kg/m)	SSWS, inclination 3,5% in the 3rd min, until exhaustion, jogging 7–8, inclination 1% every 60 sec till 5,2%, increased speed with 1 every 60 sec until exhaustion	TF 12.2(1.6) Controls 13.4(1.0)	-
Rowe, 2014	Walking	Indoor and treadmill	Energy expenditure (MET), HR	Normal and music guided	-	SSWS treadmill 110(11), music guided 118(14), SSWS indoor 114(11)
Vllasolli, 2014	Walking	-	HR, PCI	SSWS	-	-
Delussu, 2016	Walking	Indoor	VE (L/min),oxygen consumption (ml/kg/min), CO2 production (ml/kg/min), RER, HR	SSWS	TT SACH 14(4), TT 1M10 13(4)	TT SACH 117(28), TT 1M10 117(27)
Esposito, 2016	Walking	Indoor and treadmill	Oxygen consumption (VO2)	Enforced 4.5	TT ESR 13.4(0.9), BiOM 11.3(0.9), controls 12.2(1.2) 5° inclination ESR 23.1(2.5), BiOM 21.6(0.9), controls 20.9(2.2)	-
Guirao, 2016	Walking	Indoor	PCI	SSWS	-	-
Starholm, 2016	Walking	Treadmill and indoor	VO2max, oxygen uptake (ml O2/kg/min), walking economy (ml O2/kg/m), VE, RER, HR	SSWS	TF indoor 12.4(1.5), 15.8(3.4), treadmill 12.4(2.1), 15.6(2.8) Controls indoor 13.2(4), 14.6(1.9) Treadmill 13.4(4.4), 15.5(2.6)	-
Andrysek, 2017	Walking	Indoor	HR, PCI	SSWS and fast SSWS	-	-
Esposito, 2017	Walking	Treadmill and indoor	Oxygen rate (ml O2/kg/min), metabolic cost (ml O2/kg/m), HR	SSWS and 5 standardized velocities	TT 13.7(2.4), 15.8(2), 18.7(2.1), 22.7(2) SSWS indoor 19.2(3.2) Controls 9.6(1), 10.9(0.9), 12.7(1.2), 15.5(1.3), SSWS indoor 14.4(2)	TT 97(12), 105(14), 114(13), 125(15) SSWS indoor 116(17). Controls 79(14), 83(14), 88(11), 95(14), SSWS indoor 91(12)
Gardinier, 2017	Walking	Indoor	Oxygen consumption (ml/kg/min), cost of transport (J/N*m)	SSWS	TT 14.5(1.9), 14.3(1.7) Controls 13.3(0.8)	-
Gjovaag, 2017	Walking	Treadmill and indoor	Oxygen uptake (ml/kg/min), %VO2max, energy cost of walking (ml/kg/m)	SWSS, speeds 12.5% and 25% slower and faster than SWSS	TF 15.9(-) Controls 14.1(-)	-
Jarvis, 2017	Walking	Indoor	Oxygen cost (ml/kg/m), oxygen consumption (ml/kg/min)	SSWS	TT 12.3(-), TF 13.3(-), bil TF 16.2(-) Controls 11.3(-)	-
Lacraz, 2017	Walking	Treadmill	Oxygen cost (ml/kg/m), oxygen consumption (ml/kg/min), HR	SSWS	-^#^	-
Ladlow, 2017	Walking	Treadmill	MET, RPE	Enforced and with 3° and 5° inclination	-	-
Mutlu, 2017	Walking, stair ascending/descending	Indoor	6MWT, BP, HR	SSWS, 10 stairs up & down tests with/without 250g extra load	-	81(9), with extra load 85(9)
Weinert, 2017	Walking	Indoor	Oxygen consumption (-)	SSWS	-	-

n.a. not applicable;—not reported; SSWS self-selected walking speed; TT transtibial amputation, TF transfemoral amputation, KD knee disarticulation, bil bilateral; HR heart rate; BP blood pressure; PSPC pneumatic swing-phase control; NMC non-microprocessor control knee, ESR energy storing and return, BiOM bionic powered ankle–foot prosthesis 6MWT 6-min walking test; RQ respiratory quotient; RR respiratory rate (breaths/min); PCI physiological cost index ((mean HR(work)-mean HR(rest))/gait speed); %PMHR % predicted max heart rate; %HRR % heart rate reserve; EEI energy expenditure index; RER respiratory exchange ratio; MET Metabolic Equivalent of Task

* all walking speeds are converted to km/h

† result only in figures

‡ no outcome measure/numbers published

^$^ 5 different prosthetic feet

^#^results displayed only in figures, no numbers

### Meta-analysis

The heterogeneity of the study populations, test protocols and the inconsistent reporting influenced the possibilities for statistical analysis.

**Fig 2:**
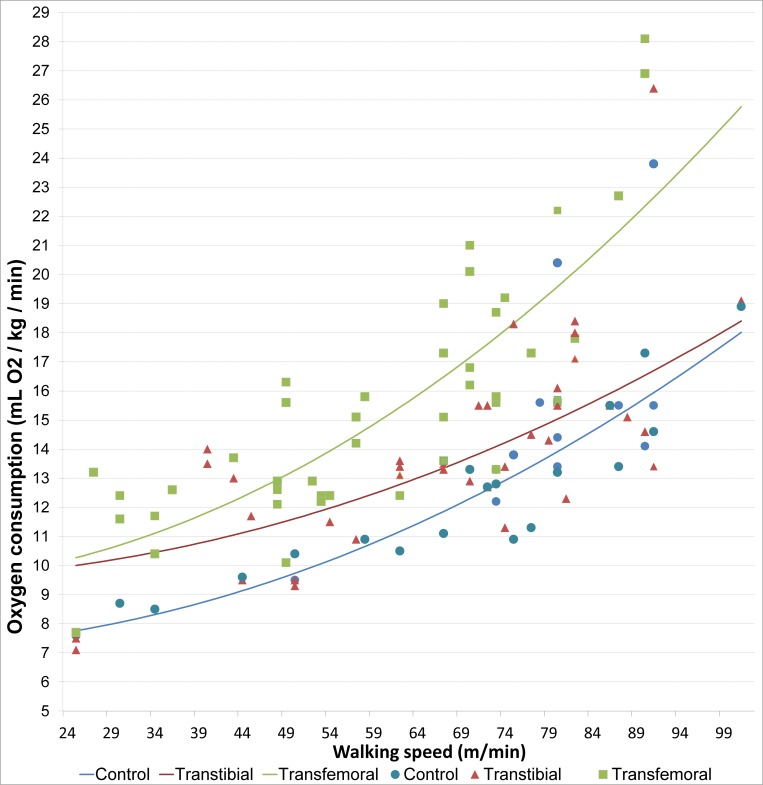
Oxygen consumption, regression lines, and the reported means for oxygen consumption (mL O_2_/kg/min) of the included studies.

When study was used as the unit of analysis, for oxygen consumption 23 studies were included in the mixed model analysis. The following significant predictors for oxygen consumption (ml O_2_/kg/min) were found: level of amputation, walking speed, and the interaction terms walking speed squared and walking speed x amputation level (Fig 2, Table 3). When adding reason of amputation to the model, 4 studies were excluded because of missing data, the model fit did not improve and the coefficients were not significant. Similar findings occurred when walking over ground vs. treadmill walking was added to the model.

**Table 3:** Estimated mean oxygen consumption (ml O_2_/kg/min) based on the meta-analysis.

**Predictor**	**Beta**	**Std. Error**	**P value**	**95% Confidence Interval**	
				Lower Bound	Upper Bound
Intercept	7.2	1.4	<0.001	4.3	10.0
Transfemoral	2.2	1.0	0.025	0.3	4.1
Transtibial	2.4	1.1	0.036	0.2	4.6
Walking speed (m/min)	-3.6 *10^−3^	3.8*10^−2^	0.925	-7.9*10^−2^	7.2*10^−2^
Walking speed^2^ (m/min)	1.1*10^−3^	3.1*10^−4^	0.001	4.8*10^−4^	1.7*10^−3^
Transfemoral * walking speed^2^	5.5*10^−4^	1.8*10^−4^	0.002	2.0*10^−4^	0.9*10^−3^
Transtibial * walking speed^2^	-1.9*10^−4^	1.9*10^−4^	0.306	-5.7*10^−4^	1.8*10^−4^

Included studies [4,16,17,22,23,25,26,32,33,41,42,46,47,51,54,56,66,67,69–71,73,74].

**Fig 3:**
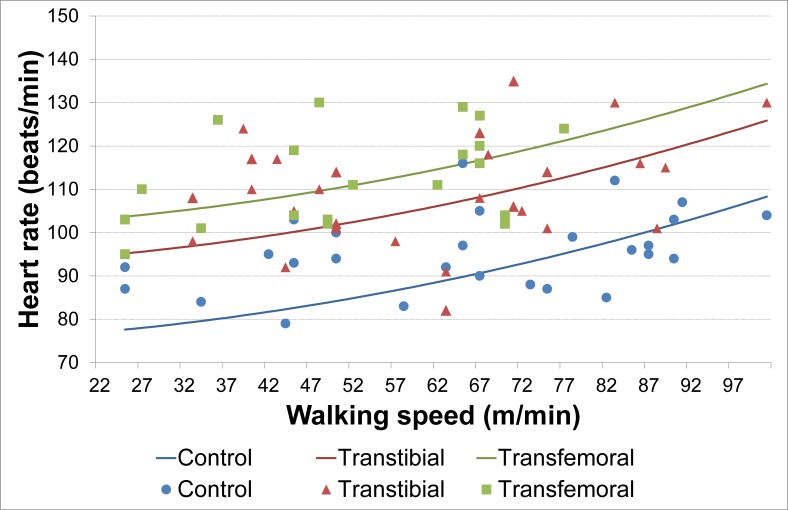
Heart rate, regression lines, and the reported means for heart rate (beats/min) of the included studies.

For heart rate, 20 studies were included in the mixed model analysis. Significant predictors for heart rate were amputation level, walking speed, and walking speed squared. No other interaction terms or other factors (e.g. reason of amputation and treadmill vs. over ground) were found to be significant (Fig 3, Table 4).

**Table 4:** Estimates mean heart rate (beats/min) based on meta-analysis.

**Predictor**	**Beta**	**Std. Error**	**P Value**	**95% Confidence Interval**	
				Lower Bound	Upper Bound
Intercept	74.5	4.8	<0.001	64.9	84.0
Transfemoral	26.1	2.2	<0.001	21.6	30.5
Transtibial	17.6	2.1	<0.001	13.4	21.8
Walking speed (m/min)	5.7*10^−2^	1.5*10^−1^	0.703	-0.2	0.3
Walking speed^2^ (m/min)	2.8*10^−3^	1.2*10^−3^	0.028	3.0*10^−4^	5.2*10^−3^

Included studies: [4,23,28–31,39,42,44,46,47,55,56,63,65,67–69,73,77].

## Discussion

Sixty-one studies (reporting on 1,912 participants) were included in this systematic review and meta-analysis. A linear mixed model was used to analyse the data. It was found that reported mean oxygen consumption (ml O_2_/kg/min) was influenced by amputation level, walking speed, and the interaction terms walking speed squared and walking speed x amputation level. Multiple previous studies reported that higher metabolic costs are associated with a more proximal level of amputation [4,5,13,82]. Previous studies also found that participants with a LLA adapt their self-selected walking speed (SSWS) in order to compensate for the higher metabolic costs [4,19,43,58]. The extent to which the SSWS is reduced depends on level of amputation [4,19,58]. The effect of walking speed on metabolic costs is also found in our analysis (Fig 2). In the multilevel analysis an increase in oxygen consumption was found at higher walking speeds, with a significant difference between reported means for controls and participants with a transfemoral amputation. However, there was no significant difference between reported means for controls and participants with a transtibial amputation. This lack of difference can be due to the included studies. For example, one study [70] measured the oxygen consumption in participants with a traumatic transtibial amputation and controls at walking speeds up to >100 m/min (Fig 2). Compared with the controls, the oxygen consumption of the participants at the highest walking speed was not significantly different (19.1±2.3 ml O_2_/kg/min vs. 18.9±1.8 ml O_2_/kg/min). In the multilevel analysis an interaction effect between walking speed squared and group was found, indicating that the effect of walking speed squared was not the same for groups. This interaction effect can be related to amputation level, but also to differences between studies including differences in methodology i.e. measurement procedures, general health, body weight, height, physical condition, and or age of the participants. However source studies report too inconsistently to be able to include these confounders in the regression analyses. The curve is based on the regression coefficients of the linear mixed model analysis of the reported means in the studies. Different study designs and heterogeneity in study populations may influence the reported means and therefore the regression curve.

One of the most noteworthy findings was that reason of amputation was not a predictor in the multilevel analysis, which may be related to the limited number of studies analysing participants with a vascular amputation. Additionally, many studies did not report on reason of amputation, had a mixed group of reasons of amputation, without specifying outcomes for separate groups. When adding reason of amputation to the mixed model analysis, 4 studies were excluded and coefficients were not significant. However, some previous studies report higher metabolic costs for walking in participants with a dysvascular amputation [4,19]. One study reported that the metabolic costs (measured by oxygen consumption (mL O_2_/kg/min)) of walking in participants with a traumatic LLA at SSWS were equal to those of controls, whereas the SSWS of the LLA group was slower than that of the control group [19]. In the group of participants with a dysvascular LLA, SSWS was slower. Even when participants with a dysvascular LLA adapted their SSWS, the metabolic costs were higher [63]. However, this study included mixed groups for level of amputation. When walking faster than their SSWS, 2 studies found higher oxygen consumption for participants with a traumatic LLA compared to controls [19,58].

The other frequently used outcome measure of metabolic costs was heart rate (beats/min). Studies reported heart rate were not all the same studies included in the analysis for oxygen consumption. Therefore, a different study population is analysed (Table 2), with a total of 20 included studies. The majority of studies using this specific outcome measure did not include participants with a LLA due to PVD. Generally, measuring heart rate is more accessible compared to oxygen consumption or VO_2_max, because in most of the settings equipment for measuring oxygen consumption and/or VO_2_max is not available. Furthermore, it is questionable whether heart rate is a good predictor of metabolic costs in persons with an amputation due to vascular disease and/or DM, because they often have comorbidities such as cardiovascular disease and are likely to use medication that influences their heart rate. Significant predictors of heart rate as an outcome measure were walking speed, amputation level, and walking speed squared. Reason for amputation was not a significant predictor of heart rate.

As stated previously, the VO_2_max-test is seen as the criterion measure of physical and aerobic capacity. Only a few studies [10,16,62,69] measured (%)VO_2_max in participants with a LLA. Only one study reported the relative aerobic load of walking, measured as a percentage of the VO_2_peak [19]. For walking, the relative aerobic load was found to be higher in participants with a LLA than in controls. Another study reported that participants with a LLA who had higher levels of physical fitness (defined as the maximum oxygen uptake during exercise as a proportion of predicted maximum oxygen uptake) were more likely to walk with a prosthesis than participants with a LLA with lower levels of physical fitness [10]. Therefore, VO_2_max may be used as an indicator for walking ability and for (physical) training in persons with a LLA. Additionally, it is relevant to know an individual’s aerobic capacity in combination with the metabolic costs of daily activities, to be able to evaluate the individual strain of ADL. Possible explanations for the lack of VO_2_max-testing in persons with a LLA are comorbidities, lack of knowledge of this type of testing, lack of knowledge of the interpretation of the test results, costs, and lack of facilities.

Previous systematic reviews did not primarily aim to analyse metabolic costs of ADL in participants with a LLA [17,18]. They found no sufficient evidence for a relation between other measures of physical capacity, such as aerobic and anaerobic capacity, and walking ability. The main finding was the heterogeneity in measurement methods and outcome measures. This finding is in keeping with the results from our study. Studies included in our systematic review and meta-analysis were also heterogeneous with respect to number of participants, participant characteristics, study characteristics, statistical analyses, test protocols, and outcome measures. The heterogeneity and the poor reporting influenced our statistical analysis. It was not possible to make forest plots or use effect sizes in the meta-analysis, therefore a conventional meta-analysis was not possible. A linear mixed model analysis was performed on the means reported, with study as unit of analysis and study results of different groups and measurement conditions as repeated measures within the unit of analysis. Despite the shortcomings of the source studies we were able to identify 2 factors (amputation level and walking speed) influencing oxygen consumption and heart rate. By combining several studies a more precise estimate was found than in single studies. All studies were of observational design, were blinding participants or investigators was not possible. In 43 of the 61 studies inclusion criteria were reported, and in only 22 studies reported exclusion criteria (S1 Table), therefore it is not clear if there was a selection bias. The majority of the included studies did report the tested walking speeds and used validated equipment. Because of the limited reporting, it was not possible to analyse predictors such as age, height, and type of prosthesis (prosthetic knee/foot), because too few studies reported sufficient details. When the aforementioned predictors were added to the statistical model, the number of studies available for analysis decreased considerably, thereby preventing further analysis. Consequently, no conclusions could be drawn from these predictors.

Of the included 61 studies, 3 studies reported on metabolic costs for walking stairs [29,30,80]. One of these studies compared heart rate for walking stairs with and without extra weight in participants with LLA and found that in both situations heart rate increased [80]. The other 2 studies measured metabolic costs (kcal/min) and heart rate [29,30]. They found a significant difference for metabolic costs (kcal/min), but no significant difference in heart rate for ascending stairs compared to controls. One other study measured different walking conditions outdoors (asphalt, mown lawn and high grass) in high functioning participants with traumatic transtibial amputation [24]. They report significant increase in oxygen consumption (ml O_2_/kg/min) and heart rate for walking in high grass, no significant differences for the other 2 conditions were found. As previously mentioned, the rehabilitation of persons with a LLA is not only aimed at walking with prosthesis, but also at regaining functional capacity and independency with regard to ADL. However, limited data are available on metabolic costs of other activities than walking with a prosthesis.

### Study limitations

The results of this systematic review and meta-analysis were dependent on the quality of research of the available studies. The included studies reported poorly on details of the study populations and potential predictors of metabolic costs in participants with a LLA. Because of the heterogeneity and the limited reporting, the mixed model analysis was performed with reported means, with study as unit of analysis. Furthermore, the studies included relatively healthy participants and, as mentioned before, data on metabolic costs for other ADL than walking is very limited.

## Future research

Further research on the metabolic costs of walking, and daily activities in persons with a LLA is relevant in order to gather data that will help with setting functional goals, optimizing individual training, and evaluating LLA-rehabilitation. Study populations and subgroups should be described with sufficient detail regarding reason for amputation, age, gender, and level of amputation to improve generalizability. The inclusion of participants with different levels of amputation in a mixed group should be avoided.

From a clinical perspective, it is important to assess the metabolic costs of ADL and the physical capacity of persons with a LLA in order to optimize their rehabilitation program, train them at an optimal walking speed, improve their potential to walk with a prosthesis, and help them regain functional capacity with regard to ADL.

## Conclusion

In general oxygen consumption and heart rate for persons with transtibial and transfemoral amputation while walking are higher than for controls. A higher walking speed is associated with a higher oxygen consumption and this increase was stronger for persons with a transfemoral amputation compared to controls. Source studies report inconsistently, therefore it is not possible to include other possible confounders in the analyses such as age and cause of amputation.

Limited information is available on metabolic costs of other activities than walking. The quality of the included studies was low; therefore, the results of this systematic review should be regarded with some caution.

## Supporting information

S1 Table AHRQ Cross-Sectional/Prevalence study quality assessment checklist. Y yes, N no, N.A. not applicable, ? not specified/unknown. *1. Was the source of information for the reported outcome measurements mentioned? 2.Were inclusion criteria reported? 3. Were exclusion criteria reported? 4. Was the timeframe of recruitment reported? 5. Were subjects consecutively recruited or population-based? 6. Were evaluators of subjective components masked to other aspects of the subjects? 7. Have any assessments been undertaken for quality assurance purposes (test/retest of primary outcome measurements)? 8. Was the used equipment validated or were there references to validation in previous publications? 9. Were all participants included in the analysis? 10.Was confounding assessed and/or controlled for? 11. Were missing data reported? 12. Were patient response rate and completeness of data collection reported? 13. Were follow-up, incomplete data, or loss to follow-up reported? 14. Was tested walking speed reported?
(DOCX)

S2 Table Frequencies of the reported outcome variables. HR heart rate; PCI physiological cost index; %HRR % heart rate reserve; RER respiratory exchange ratio; RR respiratory rate; BP blood pressure; METs metabolic equivalent of task; RQ respiratory quotient; EEI energy expenditure index.
(DOCX)

S1 File Details of the full search strategy. (DOCX)

S2 File PRISMA checklist. (DOCX)

S3 File PROSPERO. (PDF)

S4 File Data oxygen consumption. (PDF)

S5 File Data heart rate. (PDF)

## Abbreviations

LLA: lower limb amputation
ADL: activities of daily living
SSWS: self-selected walking speed
PVD: peripheral vascular disease
DM: diabetes mellitus
