# Supplementary material for: Metabolic costs of activities of daily living in persons with a lower limb amputation: A systematic review and meta-analysis
Source: PLoS One. 2019 Mar 20;14(3):e0213256. doi: 10.1371/journal.pone.0213256 (PMC6426184; doi:10.1371/journal.pone.0213256)
Supplement: S1 Table — Y yes, N no, N.A. not applicable, ? not specified/unknown. *1. Was the source of information for the reported outcome measurements mentioned? 2.Were inclusion criteria reported? 3. Were exclusion criteria reported? 4. Was the timeframe of recruitment reported? 5. Were subjects consecutively recruited or population-based? 6. Were evaluators of subjective components masked to other aspects of the subjects? 7. Have any assessments been undertaken for quality assurance purposes (test/retest of primary outcome measurements)? 8. Was the used equipment validated or were there references to validation in previous publications? 9. Were all participants included in the analysis? 10.Was confounding assessed and/or controlled for? 11. Were missing data reported? 12. Were patient response rate and completeness of data collection reported? 13. Were follow-up, incomplete data, or loss to follow-up reported? 14. Was tested walking speed reported? (DOCX) [file pone.0213256.s001.docx]

**S** **2. AHRQ Cross-Sectional/Prevalence Study Quality Assessment checklist**

| Quality Item*  Study | 1 | 2 | 3 | 4 | 5 | 6 | 7 | 8 | 9 | 10 | 11 | 12 | 13 | 14 | Total |  |
| --- | --- | --- | --- | --- | --- | --- | --- | --- | --- | --- | --- | --- | --- | --- | --- | --- |
| Ganguli, 1973 | Y | N | N | N | Y | N | N | Y | Y | N | N.A. | Y | N.A. | Y | 6 |  |
| James, 1973 | Y | ? | ? | ? | ? | N | N | Y | Y | N | N.A. | Y | N.A. | Y | 5 |  |
| Ganguli, 1974 | Y | N | N | N | Y | N | N | Y | Y | N | N.A. | Y | N.A. | Y | 6 |  |
| Ganguli, 1975 | Y | N | N | N | Y | N | N | Y | Y | N | N.A. | Y | N.A. | Y | 6 |  |
| Waters, 1976 | Y | Y | N | N | ? | N | N | Y | Y | N | N.A. | Y | N.A. | Y | 6 |  |
| Huang, 1979 | Y | N | N | N | N | N | N | Y | Y | N | N.A. | Y | N.A. | ? | 4 |  |
| Pagliarulo, 1979 | Y | N | N | N | ? | N | N | Y | Y | N | N.A. | Y | N.A. | Y | 5 |  |
| DuBow, 1983 | Y | Y | ? | N | Y | N | Y | Y | Y | N | N.A. | Y | N.A. | Y | 8 |  |
| Nowroozi, 1983 | Y | Y | N | N | Y | N | N | Y | Y | N | N.A. | Y | N.A. | Y | 7 |  |
| Isakov, 1985 | Y | N | N | N | ? | N | N | Y | Y | N | N.A. | Y | N.A. | Y | 5 |  |
| Pinzur, 1992 | Y | N | N | N | ? | N | N | Y | Y | N | N.A. | Y | N.A. | Y | 5 |  |
| Gailey, 1993 | Y | Y | N | N | ? | N | N | Y | Y | N | N.A. | Y | N.A. | Y | 6 |  |
| Jaegers, 1993 | Y | N | N | N | N | N | N | Y | Y | N | ? | Y | N.A. | Y | 5 |  |
| Boonstra, 1994 | Y | Y | N | N | Y | Y | N | Y | Y | N | N.A. | Y | N.A. | Y | 8 |  |
| Gailey, 1994 | Y | Y | N | N | ? | N | N | Y | Y | N | N.A. | Y | N.A. | Y | 6 |  |
| Torburn, 1995 | Y | Y | N | N | Y | N | N | Y | Y | N | N.A. | Y | N.A. | Y | 7 |  |
| Hoffman, 1997 | Y | N | N | N | N | N | N | Y | Y | N | N.A. | Y | N.A. | Y | 5 |  |
| Chin, 2002 | Y | Y | N | N | Y | N | N | N.A. | Y | N | N.A. | Y | N.A. | Y | 6 |  |
| Schmalz, 2002 | Y | N | Y | N | ? | N | N | Y | Y | N | N.A. | Y | N.A. | Y | 6 |  |
| Bussmann, 2004 | Y | Y | Y | N | Y | N | Y | Y | Y | N | N | Y | Y | Y | 10 |  |
| Datta,2005 | Y | ? | ? | ? | ? | N | N | Y | Y | N | N.A. | Y | N.A. | Y | 5 |  |
| Chin, 2006 | Y | Y | N | N | N | N | N | Y | Y | N | N.A. | Y | N.A. | Y | 6 |  |
| Chin, 2006 | Y | Y | N | N | Y | N | N | N.A. | Y | N | N.A. | Y | N.A. | Y | 6 |  |
| Paysant, 2006 | Y | Y | N | Y | ? | N | N | Y | Y | N | N.A. | Y | N.A. | Y | 7 |  |
| Hagberg, 2007 | Y | Y | N | N | Y | N | N | ? | Y | N | N.A. | Y | N.A. | N | 5 |  |
| Seymour, 2007 | Y | Y | N | N | Y | N | N | Y | Y | N | N.A. | Y | N.A. | Y | 7 |  |
| Bussmann, 2008 | Y | Y | Y | N | Y | N | N | N.A. | N | N | N | Y | N.A. | Y | 6 |  |
| Genin, 2008 | Y | N | N | N | ? | N | N | Y | Y | N | N.A. | Y | N.A. | Y | 5 |  |
| Kaufman, 2008 | Y | Y | Y | N | Y | N | N | Y | Y | N | N.A. | Y | N.A. | Y | 8 |  |
| Traballesi, 2008 | Y | Y | N | N | Y | N | N | Y | Y | N | N.A. | Y | N.A. | Y | 7 |  |
| Wright, 2008 | Y | Y | N | N | Y | N | N | Y | Y | N | N.A. | Y | N.A. | Y | 7 |  |
| Hamamura, 2009 | Y | Y | Y | N | Y | N | N | N.A. | Y | N | N.A. | Y | N.A. | Y | 7 |  |
| Houdijk, 2009 | Y | N | N | N | ? | N | N | Y | Y | N | N.A. | Y | N.A. | Y | 5 |  |
| Tekin, 2009 | Y | Y | Y | Y | Y | N | N | Y | Y | N | N.A. | Y | N.A. | Y | 9 |  |
| Goktepe, 2010 | Y | Y | Y | N | Y | N | N | Y | Y | N | N.A. | Y | N.A. | Y | 8 |  |
| Andrysek, 2011 | Y | Y | N | N | Y | N.A. | N | Y | Y | N | N.A. | Y | N | Y | 7 |  |
| Hagberg, 2011 | Y | Y | N | N | Y | N | Y | ? | Y | N | N.A. | Y | N.A. | ? | 6 |  |
| Kark, 2011 | Y | N | Y | N | Y | N | N | Y | Y | N | N.A. | Y | N.A. | Y | 7 |  |
| Mohanty, 2012 | Y | Y | Y | N | Y | N | N | Y | Y | N | N.A. | Y | N.A. | Y | 8 |  |
| Schnall, 2012 | Y | Y | N | N | Y | N | N | Y | Y | N | N.A. | Y | N.A. | Y | 7 |  |
| Sokhangoei, 2013 | Y | Y | N | N | Y | N | N | Y | Y | N | N.A. | Y | N.A. | ? | 6 |  |
| Wezenberg, 2013 | Y | Y | Y | N | Y | N | N | Y | Y | N | N.A. | Y | N.A. | Y | 8 |  |
| Bell, 2014 | Y | Y | Y | Y | Y | N.A. | N | Y | Y | N | N.A. | Y | N.A. | Y | 9 |  |
| Erjavec, 2014 | Y | Y | Y | Y | Y | N | N | N | N | N | Y | Y | N.A. | Y | 8 |  |
| Esposito, 2014 | Y | Y | Y | N | Y | N | N | Y | Y | N | N.A. | Y | N.A. | Y | 8 |  |
| Gjovaag, 2014 | Y | N | N | N | N | N | N | Y | Y | N | N.A. | Y | N.A. | Y | 5 |  |
| Rowe, 2014 | Y | Y | Y | N | Y | N | N | Y | Y | N | N.A. | Y | N.A. | Y | 8 |  |
| Vllasolli, 2014 | Y | Y | N | N | Y | N | N | Y | Y | N | N.A. | Y | N.A. | ? | 6 |  |
| Delussu, 2016 | Y | Y | N | N | ? | N.A. | N | N | Y | N | N.A. | N | N.A. | Y | 4 |  |
| Esposito, 2016 | Y | N | N | N | ? | N.A. | N | N | N | N | N | Y | N.A. | Y | 3 |  |
| Guirao,2016 | Y | Y | Y | Y | Y | N.A. | N | Y | Y | N | N.A. | Y | Y | Y | 10 |  |
| Starholm, 2016 | Y | Y | N | N | ? | N.A. | N | Y | Y | N | N.A. | Y | N.A. | Y | 6 |  |
| Andrysek, 2017 | Y | Y | N | N | Y | N.A. | N | N | Y | N | N | Y | N.A. | Y | 6 |  |
| Esposito, 2017 | Y | Y | Y | N | Y | N | N | Y | Y | N | N.A. | Y | N.A. | Y | 8 |  |
| Gardinier, 2017 | Y | Y | Y | Y | Y | N | N | Y | N | N | N.A. | Y | N.A. | Y | 8 |  |
| Gjovaag, 2017 | N | N | Y | N | ? | N | N | Y | Y | N | N.A. | Y | N.A. | Y | 5 |  |
| Jarvis, 2017 | Y | Y | N | Y | Y | N | N | Y | Y | N | N.A. | Y | N.A. | Y | 8 |  |
| Lacraz, 2017 | Y | Y | Y | Y | Y | N.A. | N | N | Y | N | N.A. | Y | N | Y | 8 |  |
| Ladlow, 2017 | Y | Y | Y | N | Y | N | N | Y | Y | N | N.A. | Y | N.A. | Y | 8 |  |
| Mutlu, 2017 | Y | Y | Y | N | Y | N | N | Y | Y | N | N.A. | Y | N.A. | Y | 8 |  |
| Weinert, 2017 | Y | Y | Y | N | Y | N.A. | N | Y | Y | N | N.A. | Y | N.A. | Y | 8 |  |
| Total | 60 | 43 | 22 | 8 | 40 | 1 | 3 | 50 | 57 | 0 | 1 | 60 | 2 | 56 |  |  |

Y yes, N no, N.A. not applicable, ? not specified/unknown.

*1. Was the source of information for the reported outcome measurements mentioned? 2.Were inclusion criteria reported? 3. Were exclusion criteria reported? 4. Was the timeframe of recruitment reported? 5. Were subjects consecutively recruited or population-based? 6. Were evaluators of subjective components masked to other aspects of the subjects? 7. Have any assessments been undertaken for quality assurance purposes (test/retest of primary outcome measurements)? 8. Was the used equipment validated or were there references to validation in previous publications? 9. Were all participants included in the analysis? 10.Was confounding assessed and/or controlled for? 11. Were missing data reported? 12. Were patient response rate and completeness of data collection reported? 13. Were follow-up, incomplete data, or loss to follow-up reported? 14. Was tested walking speed reported?
