# Supplementary material for: Metabolic costs of activities of daily living in persons with a lower limb amputation: A systematic review and meta-analysis
Source: PLoS One. 2019 Mar 20;14(3):e0213256. doi: 10.1371/journal.pone.0213256 (PMC6426184; doi:10.1371/journal.pone.0213256)
Supplement: S2 Table — HR heart rate; PCI physiological cost index; %HRR % heart rate reserve; RER respiratory exchange ratio; RR respiratory rate; BP blood pressure; METs metabolic equivalent of task; RQ respiratory quotient; EEI energy expenditure index. (DOCX) [file pone.0213256.s002.docx]

| **S 3. Reported outcome measures in methods**   \| **Main outcome variables** \| **Studies** (n) \| \| --- \| --- \| \| HR (beats/min) \| 39 \| \| VO_2_/oxygen uptake / oxygen consumption  (ml/kg/min, L/min) \| 35 \| \| Oxygen cost (ml/kg/m) \| 16 \| \| PCI \| 8 \| \| (%)VO_2_max \| 6 \| \| VO_2_ peak \| 1 \| \| Peak HR \| 3 \| \| % HRR \| 2 \| \| Energy expenditure (kcal/min) \| 2 \| \| Energy expenditure (J/s/kg) \| 1 \| \| Energy expenditure (kcal/kg/km and kcal/km) \| 1 \| \| Energy expenditure (cal/min/kg) \| 1 \| \| Gross cost / net cost (J/kg/m) \| 1 \| \| RER \| 3 \| \| Metabolic energy consumption (J/kg/s)  Metabolic energy cost (J/kg/m) \| 1 \| \| Basal energy expenditure  energy cost (cal/ft/kg)  oxygen consumption (ml/ft/kg) \| 1 \| \| Blood lactate \| 1 \| \| RR \| 1 \| \| BP \| 2 \| \| METs \| 1 \| \| Relative energy cost (%), \| 1 \| \| RQ \| 2 \| \| EEI (ml/kg/min) \| 1 \| |  |
| --- | --- | --- | --- | --- | --- | --- | --- | --- | --- | --- | --- | --- | --- | --- | --- | --- | --- | --- | --- | --- | --- | --- | --- | --- | --- | --- | --- | --- | --- | --- | --- | --- | --- | --- | --- | --- | --- | --- | --- | --- | --- | --- | --- | --- | --- | --- | --- | --- | --- |
|  |  |
