# Supplementary material for: Metabolic costs of activities of daily living in persons with a lower limb amputation: A systematic review and meta-analysis
Source: PLoS One. 2019 Mar 20;14(3):e0213256. doi: 10.1371/journal.pone.0213256 (PMC6426184; doi:10.1371/journal.pone.0213256)
Supplement: S1 File — (DOCX) [file pone.0213256.s003.docx]

**S 1. Details of the full search strategy**

Pubmed:

| (("Lower Extremity"[Mesh]) AND "Amputation"[Mesh] OR Syme amputation[tw] OR above ankle amputation[tw] OR transtibial amputation[tw] OR knee-exarticulation[tw] OR knee exarticulation[tw] OR knee disarticulation [tw] OR knee-disarticulation [tw] OR above knee amputation[tw] OR transfemoral amputation[tw] OR hip exarticulation[tw] OR hip-exarticulation[tw] OR hip disarticulation[tw] OR hip-disarticulation[tw] OR Syme amputee*[tw] OR above ankle amputee*[tw] OR transtibial amputee*[tw] OR above knee amputee*[tw] OR transfemoral amputee*[tw] OR lower extremity amputation[tw] OR lower extremity amputee[tw] OR lower limb amputation[tw] OR lower limb amputee[tw] OR leg amputation[tw] OR below-knee amputation [tw] OR below knee amputation[tw] OR Lower extremity amputee*[tw] OR lower limb amputee*[tw] OR leg amputee*[tw] OR below-knee-amputee*[tw] OR below knee amputee*[tw] OR lower limb prosthe*[tw] OR leg prosthe*[tw] OR artificial leg[tw]) |
| --- |
| AND (("Metabolism"[Mesh] OR energy[tw] OR metabol*[tw] OR aerobic[tw] OR net value[tw] OR joule*[tw] OR calor*[tw] OR pci[tw] OR physiological cost index[tw] OR anaerobic[tw] OR energetic [tw]))) |
| AND (("Activities of Daily Living"[Mesh] OR Activities of Daily Living[tw] OR adl[tw] OR (("Walking"[Mesh]) OR "Monitoring, Ambulatory"[Mesh]) OR "Gait"[Mesh] OR walk*[tw] OR gait[tw] OR "Bicycling"[Mesh] OR bicycl*[tw] OR stair[tw] OR dress*[tw] OR cook*[tw] OR ambulant[tw] OR ambulation[tw] OR self-care[tw] OR self care[tw] OR home activit*[tw] OR drive[tw] OR driving[tw] OR transfer[tw] OR garden*[tw] OR human activit*[tw] OR run*[tw]))) |

Embase:

| ‘leg amputation’/exp OR ‘leg prosthesis’/exp OR 'leg amputation':ti,ab OR 'transtibial amputation':ti,ab OR 'syme amputation':ti,ab OR 'above ankle amputation':ti,ab OR 'knee amputation':ti,ab OR 'transfemoral amputation':ti,ab OR 'hip exarticulation':ti,ab OR 'hip disarticulation prosthesis':ti,ab OR 'leg amputation' OR 'transtibial amputation' OR 'syme amputation' OR 'above ankle amputation' OR 'knee amputation' OR 'transfemoral amputation' OR 'hip exarticulation' OR 'hip disarticulation prosthesis' OR ‘amputees’ OR amputee:ti,ab |
| --- |
| AND 'metabolic rate'/exp OR 'metabolism'/exp OR 'energy cost'/exp OR metabolism:ab,ti OR energy:ab,ti OR metabolic:ab,ti OR 'metabolic rate':ab,ti OR 'metabolism':ab,ti OR 'calorie'/exp OR 'calorie':ab,ti OR calorie OR 'metabolic equivalent'/exp OR 'metabolic equivalent':ab,ti OR 'met value':ab,ti OR 'physiological cost index':ab,ti OR 'pci':ab,ti OR metabolism OR energy, OR metabolic OR 'met value' OR 'physiological cost index' OR 'pci' |
| AND ‘human activities’/exp OR ‘daily life activity’/exp OR ‘human activities’:ab,ti OR ‘daily life activity’:ab,ti OR ‘walking’/exp OR ‘walking’:ab,ti OR ‘gait’:ab,ti OR ‘physical acitity’/exp OR ‘physical activity’:ab,ti OR ‘self care’/exp OR ‘self care’:ab,ti OR ‘running’:ab,ti |

Cinahl:

| (MH "Lower Extremity+") OR "lower extremity" AND (MH "Amputation+") OR "amputation" OR (MH "Below-Knee Amputation") OR (MH "Above-Knee Amputation") OR (MH "Amputation, Traumatic") OR "lower limb amputation" OR MH "Leg") OR "leg" OR (MH "Limb Prosthesis") MH "Below-Knee Amputation") OR (MH "Above-Knee Amputation") OR (MH "Amputation, Traumatic") OR "knee amputation" OR (MH "Disarticulation") OR "transtibial amputation" OR "above knee amputation" OR "hip disarticulation" OR (MH "Limb Prosthesis") OR "artificial leg" |
| --- |
| AND (MH "Metabolism+") OR "metabolism" OR (MH "Basal Metabolism+") OR (MH "Energy Metabolism+") OR (MH "Energy Conservation, Metabolic") OR "energy" OR "energy cost" OR "met value" OR "metabolic equivalent" OR "pci" OR "physiological cost index" OR (MH "Basal Metabolic Rate") OR "calorie" OR "metabolic rate" |
| AND (MH "Activities of Daily Living+") OR "adl" OR (MH "Grooming+") OR "grooming" OR (MH "Oral Hygiene+") OR (MH "Home Rehabilitation+") OR (MH "Occupational Therapy+") OR (MH "Physical Therapy+") OR (MH "Rehabilitation, Psychosocial+") OR (MH "Rehabilitation, Pulmonary+") OR (MH "Running+") OR "running" OR (MH "Walking+") OR "walking" OR (MH "Human Activities+") OR "human activities" OR (MH "Gait+") OR "gait" OR (MH "Self Care+") OR "self care" OR (MH "Cycling") OR "cycling" OR "biclycling" OR (MH "Stair Climbing") OR "stairs" OR "climbing stairs" |

PsychINFO:

| DE "Amputation" OR DE "Phantom Limbs" OR DE "Prostheses" |
| --- |
| AND (DE "Metabolic Rates" OR DE "Metabolism" OR DE "Basal Metabolism" OR DE "Biosynthesis" OR DE "Carbohydrate Metabolism" OR DE "Catabolism" OR DE "Lipid Metabolism" OR DE "Metabolites" OR DE "Protein Metabolism") OR (DE "Energy Expenditure")) OR (DE "Calories") ) OR metabolic equivalent OR physiological cost index OR calorie OR energy |
| AND (DE "Activities of Daily Living") OR (DE "Running")) OR (DE "Walking")) OR (DE "Gait")) OR (DE "Physical Activity" OR DE "Exercise")) AND (DE "Self Care Skills" OR DE "Ability" OR DE "Activities of Daily Living" OR DE "Daily Activities" OR DE "Hygiene" OR DE "Independent Living Programs" OR DE "Rehabilitation" OR DE "Skill Learning") |

CENTRAL:

| [Amputation] or [amputees] OR [artificial limb] or amput* or prosthes* or artificial limb or transtibial amputat* or through knee amputat* or transfemoral amput* or Syme amputation or above ankle amputation or knee-exarticulation or knee exarticulation or knee disarticulation or knee-disarticulation or above knee amputation or hip exarticulation or hip-exarticulation or hip disarticulation or hip-disarticulation or Syme amputee* or above ankle amputee* or transtibial amputee* or above knee amputee* or lower extremity amputation or lower extremity amputee or lower limb amputation or lower limb amputee or leg amputation or below knee amputation or Lower extremity amputee* or lower limb amputee* or leg amputee* or below knee amputee* or lower limb prosthe* or leg prosthe* or artificial leg |
| --- |
| AND [energy metabolism] or [metabolism] energy OR metabol* OR aerobic OR net value OR joule* OR calor* OR pci OR physiological cost index OR anaerobic OR energetic |
| AND [Human activities] or [activities of daily living] or activities of Daily Living OR adl OR Monitoring, Ambulatory OR walk* OR gait OR Bicycling OR bicycl* OR stair OR dress* OR cook* OR ambulant OR ambulation OR self-care OR self care OR home activit* OR drive OR driving OR transfer OR garden* OR human activit* OR run* |
