# Supplementary material for: Metabolic costs of activities of daily living in persons with a lower limb amputation: A systematic review and meta-analysis
Source: PLoS One. 2019 Mar 20;14(3):e0213256. doi: 10.1371/journal.pone.0213256 (PMC6426184; doi:10.1371/journal.pone.0213256)
Supplement: S3 File — (PDF) [file pone.0213256.s005.pdf]

Systematic review

Please complete all mandatory fields below (marked with an asterisk *) and as many of the non-mandatory fields as you can then click *Submit* to submit your registration. You don't need to complete everything in one go, this record will appear in your *My PROSPERO* section of the web site and you can continue to edit it until you are ready to submit. Click *Show help* below or click on the icon
to see guidance on completing each section.
This record cannot be edited because it has been rejected

1. * Review title.

Give the working title of the review, for example the one used for obtaining funding. Ideally the title should state succinctly the interventions or exposures being reviewed and the associated health or social problems. Where appropriate, the title should use the PI(E)COS structure to contain information on the Participants, Intervention (or Exposure) and Comparison groups, the Outcomes to be measured and Study designs to be included.
Metabolic costs of activities of daily living in subjects with a lower limb amputation: a systematic review and meta-analysis.

2. Original language title.

For reviews in languages other than English, this field should be used to enter the title in the language of the review. This will be displayed together with the English language title.

3. * Anticipated or actual start date.

Give the date when the systematic review commenced, or is expected to commence.
01/03/2016

4. * Anticipated completion date.

Give the date by which the review is expected to be completed.
31/05/2018

5. * Stage of review at time of this submission.

Indicate the stage of progress of the review by ticking the relevant Started and Completed boxes. Additional information may be added in the free text box provided.
Please note: Reviews that have progressed beyond the point of completing data extraction at the time of initial registration are not eligible for inclusion in PROSPERO. Should evidence of incorrect status and/or completion date being supplied at the time of submission come to light, the content of the PROSPERO record will be removed leaving only the title and named contact details and a statement that inaccuracies in the stage of the review date had been identified.
This field should be updated when any amendments are made to a published record and on completion and publication of the review.

The review has not yet started: No

Review stage	Started	Completed
Preliminary searches	Yes	Yes
Piloting of the study selection process	Yes	Yes
Formal screening of search results against eligibility criteria	Yes	Yes
Data extraction	Yes	Yes
Risk of bias (quality) assessment	Yes	Yes
Data analysis	Yes	Yes

Provide any other relevant information about the stage of the review here (e.g. Funded proposal, protocol not yet finalised).

6. * Named contact.

The named contact acts as the guarantor for the accuracy of the information presented in the register record.
Ms van Schaik

Email salutation (e.g. "Dr Smith" or "Joanne") for correspondence:

7. * Named contact email.

Give the electronic mail address of the named contact.
l.van.schaik@umcg.nl

8. Named contact address

Give the full postal address for the named contact.
UMCG Rehabilitation Medicine, Hanzeplein 1, 9713 GZ Groningen, Postbus 30.001, 9700 RB Groningen, Netherlands.

9. Named contact phone number.

Give the telephone number for the named contact, including international dialling code.
0031622089854

10. * Organisational affiliation of the review.

Full title of the organisational affiliations for this review and website address if available. This field may be completed as 'None' if the review is not affiliated to any organisation.
Universitair medisch centrum Groningen (UMCG)

Organisation web address:

11. Review team members and their organisational affiliations.

Give the title, first name, last name and the organisational affiliations of each member of the review team. Affiliation refers to groups or organisations to which review team members belong.

Ms Loeke van Schaik. UMCG Center for Rehabilitation
Professor Jan Geertzen. UMCG Center for Rehabilitation
Dr Rienk Dekker. UMCG Center for Rehabilitation
Professor Pieter Dijkstra. UMCG Center for Rehabilitation

12. * Funding sources/sponsors.

Give details of the individuals, organizations, groups or other legal entities who take responsibility for initiating, managing, sponsoring and/or financing the review. Include any unique identification numbers assigned to the review by the individuals or bodies listed.

None.

13. * Conflicts of interest.

List any conditions that could lead to actual or perceived undue influence on judgements concerning the main topic investigated in the review.

None

14. Collaborators.

Give the name and affiliation of any individuals or organisations who are working on the review but who are not listed as review team members.

15. * Review question.

State the question(s) to be addressed by the review, clearly and precisely. Review questions may be specific or broad. It may be appropriate to break very broad questions down into a series of related more specific questions. Questions may be framed or refined using PI(E)COS where relevant.

What are the metabolic costs of walking in lower-extremity amputees?
Which methods and outcome variables are used to evaluate metabolic cost?
What is the effect of the level and reason of amputation on metabolic costs?

16. * Searches.

Give details of the sources to be searched, search dates (from and to), and any restrictions (e.g. language or publication period). The full search strategy is not required, but may be supplied as a link or attachment.

A literature search was performed using PubMed, EMBASE, CINAHL, PsycINFO and Cochrane Central Register of Controlled Trials (CENTRAL). There were no language or date restrictions. Different terms for lower-limb amputation (LLA) were used, combined with all known terms for metabolic costs, and physical activities, and/or activities of daily living.

17. URL to search strategy.

Give a link to the search strategy or an example of a search strategy for a specific database if available (including the keywords that will be used in the search strategies).

Alternatively, upload your search strategy to CRD in pdf format. Please note that by doing so you are consenting to the file being made publicly accessible.

Yes I give permission for this file to be made publicly available

18. * Condition or domain being studied.

Give a short description of the disease, condition or healthcare domain being studied. This could include health and wellbeing outcomes.

People with lower-limb amputation.

19. * Participants/population.

Give summary criteria for the participants or populations being studied by the review. The preferred format includes details of both inclusion and exclusion criteria.

Inclusion criteria: studies involved adult subjects with a lower-limb amputation, one or more outcome measures of energy costs / metabolic costs, involving a physical activity or an activity of daily living.
Exclusion criteria: editorial, expert opinion, paper, comments, reviews, off-topic studies, toe or mid-foot

amputations, amputations other than lower-extremity amputation or endoprosthesis, studies with children, animals or models.
If 50 studies remain, studies with a total population 10, and articles in French or German will be excluded.

20. * Intervention(s), exposure(s).

Give full and clear descriptions or definitions of the nature of the interventions or the exposures to be reviewed.
Exposure: walking (indoor, outdoor, or treadmill).

21. * Comparator(s)/control.

Where relevant, give details of the alternatives against which the main subject/topic of the review will be compared (e.g. another intervention or a non-exposed control group). The preferred format includes details of both inclusion and exclusion criteria.
Controls (non amputee) transtibial, transfemoral and different walking speeds.

22. * Types of study to be included.

Give details of the types of study (study designs) eligible for inclusion in the review. If there are no restrictions on the types of study design eligible for inclusion, or certain study types are excluded, this should be stated. The preferred format includes details of both inclusion and exclusion criteria.
We will include cohort and observational studies to evaluate what is known about metabolic costs in people with a lower-limb amputation.

23. Context.

Give summary details of the setting and other relevant characteristics which help define the inclusion or exclusion criteria.

24. * Primary outcome(s).

Give the pre-specified primary (most important) outcomes of the review, including details of how the outcome is defined and measured and when these measurement are made, if these are part of the review inclusion criteria.
One or more outcome measures of energy costs / metabolic costs, for example VO2max, VO2, or heart rate. Involving a physical activity or an activity of daily living. This includes walking indoors or outdoors (flat or uneven), obstacles, walking up stairs, etc.

Timing and effect measures

25. * Secondary outcome(s).

List the pre-specified secondary (additional) outcomes of the review, with a similar level of detail to that required for primary outcomes. Where there are no secondary outcomes please state ‘None’ or ‘Not applicable’ as appropriate to the review
None.

Timing and effect measures

26. Data extraction (selection and coding).

Give the procedure for selecting studies for the review and extracting data, including the number of researchers involved and how discrepancies will be resolved. List the data to be extracted.
Titles and/or abstracts of studies retrieved using the search strategy and those from additional sources will be screened independently by two review authors to identify studies that potentially meet the inclusion criteria. The full texts of these potentially eligible studies will be retrieved and independently assessed for eligibility by two review team members. Where there is no consensus, articles will be included.

A standardised, pre-piloted form will be used to extract data from the included studies for assessment of study quality and evidence synthesis. Extracted information will include: study setting; study population and participant demographics; details of the intervention and control conditions; study methodology; recruitment and study completion rates; outcomes and times of measurement; information for assessment of the risk of bias.
Two review authors will extract data independently, discrepancies will be identified and resolved through discussion (with a third author where necessary). Missing data will be requested from study authors.

27. * Risk of bias (quality) assessment.

State whether and how risk of bias will be assessed (including the number of researchers involved and how discrepancies will be resolved), how the quality of individual studies will be assessed, and whether and how this will influence the planned synthesis.
For quality assessment we will use the (slightly) adapted AHRQ Cross-Sectional/Prevalence Study Quality Assessment checklist.

28. * Strategy for data synthesis.

Give the planned general approach to synthesis, e.g. whether aggregate or individual participant data will be used and whether a quantitative or narrative (descriptive) synthesis is planned. It is acceptable to state that a quantitative synthesis will be used if the included studies are sufficiently homogenous.
If an appropriate number of studies is available, we will perform a meta regression to explore variables related to metabolic costs.

29. * Analysis of subgroups or subsets.

Give details of any plans for the separate presentation, exploration or analysis of different types of participants (e.g. by age, disease status, ethnicity, socioeconomic status, presence or absence or co-morbidities); different types of intervention (e.g. drug dose, presence or absence of particular components of intervention); different settings (e.g. country, acute or primary care sector, professional or family care); or different types of study (e.g. randomised or non-randomised).
Variables related to metabolic costs, if possible.

30. * Type and method of review.

Select the type of review and the review method from the lists below. Select the health area(s) of interest for your review.

Type of review

Cost effectiveness
No

Diagnostic
No

Epidemiologic
Yes

Individual patient data (IPD) meta-analysis
No

Intervention
No

Meta-analysis
Yes

Methodology
No

Network meta-analysis
No

Pre-clinical

No

Prevention
No

Prognostic
Yes

Prospective meta-analysis (PMA)
No

Qualitative synthesis
No

Review of reviews
No

Service delivery
No

Systematic review
Yes

Other
No

Wounds, injuries and accidents

Health area of the review

Alcohol/substance misuse/abuse
No
Blood and immune system
No
Cancer
No
Cardiovascular
No
Care of the elderly
No
Child health
No
Complementary therapies
No
Crime and justice
No
Dental
No
Digestive system
No
Ear, nose and throat
No
Education
No
Endocrine and metabolic disorders
No
Eye disorders
No
General interest
No
Genetics
No
Health inequalities/health equity
No
Infections and infestations

No
International development
No
Mental health and behavioural conditions
No
Musculoskeletal
No
Neurological
No
Nursing
No
Obstetrics and gynaecology
No
Oral health
No
Palliative care
No
Perioperative care
No
Physiotherapy
No
Pregnancy and childbirth
No
Public health (including social determinants of health)
No
Rehabilitation
No
Respiratory disorders
No
Service delivery
No
Skin disorders
No
Social care
No
Surgery
No
Tropical Medicine
No
Urological
No
Wounds, injuries and accidents
No
Violence and abuse
No

31. Language.

Select each language individually to add it to the list below, use the bin icon to remove any added in error.
English

There is an English language summary.

32. Country.

Select the country in which the review is being carried out from the drop down list. For multi-national collaborations select all the countries involved.
Netherlands

33. Other registration details.

Give the name of any organisation where the systematic review title or protocol is registered (such as with The Campbell Collaboration, or The Joanna Briggs Institute) together with any unique identification number assigned. (N.B. Registration details for Cochrane protocols will be automatically entered). If extracted data will be stored and made available through a repository such as the Systematic Review Data Repository (SRDR), details and a link should be included here. If none, leave blank.

34. Reference and/or URL for published protocol.

Give the citation and link for the published protocol, if there is one

Give the link to the published protocol.

Alternatively, upload your published protocol to CRD in pdf format. Please note that by doing so you are consenting to the file being made publicly accessible.

Yes I give permission for this file to be made publicly available

Please note that the information required in the PROSPERO registration form must be completed in full even if access to a protocol is given.

35. Dissemination plans.

Give brief details of plans for communicating essential messages from the review to the appropriate audiences.

Do you intend to publish the review on completion?

Yes

36. Keywords.

Give words or phrases that best describe the review. Separate keywords with a semicolon or new line. Keywords will help users find the review in the Register (the words do not appear in the public record but are included in searches). Be as specific and precise as possible. Avoid acronyms and abbreviations unless these are in wide use.

Metabolic costs
Lower limb amputation
physical activity
ADL

37. Details of any existing review of the same topic by the same authors.

Give details of earlier versions of the systematic review if an update of an existing review is being registered, including full bibliographic reference if possible.

38. * Current review status.

Review status should be updated when the review is completed and when it is published.

Please provide anticipated publication date

Review_Completed_not_published

39. Any additional information.

Provide any other information the review team feel is relevant to the registration of the review.

40. Details of final report/publication(s).

This field should be left empty until details of the completed review are available.

Give the link to the published review.
