# Supplementary material for: Metabolic costs of activities of daily living in persons with a lower limb amputation: A systematic review and meta-analysis
Source: PLoS One. 2019 Mar 20;14(3):e0213256. doi: 10.1371/journal.pone.0213256 (PMC6426184; doi:10.1371/journal.pone.0213256)
Supplement: S4 File — (PDF) [file pone.0213256.s006.pdf]

number	study	repetition within study	n	walking speed m/min
1	Bell et al 2014	1	26	77
2	Bell et al 2014	2	26	67
3	Boo tra	1	29	
4	Bussmann et al 2004	1	10	
5	Bussmann et al 2004	2	10	
6	Bussmann et al 2008	1	9	
7	Bussmann et al 2008	2	9	
8	Chin et al 2002	1	8	
9	Chin et al 2002	2	9	
10	Chin et al 2006a	1	34	
11	Chin et al 2006a	2	15	
12	Chin et al 2006b	1	14	30
13	Chin et al 2006b	2	14	50
14	Chin et al 2006b	3	14	70
15	Chin et al 2006b	4	14	90
16	Chin et al 2006b	5	4	30
17	Chin et al 2006b	6	4	50
18	Chin et al 2006b	7	4	70
19	Chin et al 2006b	8	4	90
20	Chin et al 2006b	9	4	30
21	Chin et al 2006b	10	4	50
22	Chin et al 2006b	11	4	70
23	Chin et al 2006b	12	4	90
24	Datta et al 2005	1	10	
25	Dubow et al 1983	1	6	40
26	Dubow et al 1983	2	6	45
27	Dubow et al 1983	3	8	63,3
28	Dubow et al 1983	4	8	41,7
29	Erjavec et al 2013	1	101	
30	Erjavec et al 2013	2	101	
31	Esposito et al 2014	1	13	44,4
32	Esposito et al 2014	2	13	57
33	Esposito et al 2014	3	13	72
34	Esposito et al 2014	4	13	86,4
35	Esposito et al 2014	5	13	100,8
36	Esposito et al 2014	6	13	43,8
37	Esposito et al 2014	7	13	58,2
38	Esposito et al 2014	8	13	72,6
39	Esposito et al 2014	9	13	87
40	Esposito et al 2014	10	13	101,4
41	Gailey et al 1993	1	10	33,5
42	Gailey et al 1993	2	10	67
43	Gailey et al 1993	3	10	33,5
44	Gailey et al 1993	4	10	67
45	Gailey et al 1993	5	10	33,5
46	Gailey et al 1993	6	10	67
47	Gailey et al 1994	1	39	70
48	Gailey et al 1994	2	21	75
49	Ganguli et al 1973	1	10	50

50	Ganguli et al 1973	2	10	
51	Ganguli et al 1973	3	10	
52	Ganguli et al 1973	4	10	
53	Ganguli et al 1973	5	10	
54	Ganguli et al 1973	6	16	50
55	Ganguli et al 1973	7	16	
56	Ganguli et al 1973	8	16	
57	Ganguli et al 1973	9	16	
58	Ganguli et al 1973	10	16	
59	Ganguli et al 1974	1	6	50
60	Ganguli et al 1974	2	6	67
61	Ganguli et al 1974	3	6	83
62	Ganguli et al 1974	4	6	50
63	Ganguli et al 1974	5	6	67
64	Ganguli et al 1974	6	6	83
65	Ganguli et al 1975	1	10	50
66	Ganguli et al 1975	2	10	
67	Ganguli et al 1975	3	10	
68	Ganguli et al 1975	4	10	
69	Ganguli et al 1975	5	10	
70	Ganguli et al 1975	6	10	50
71	Ganguli et al 1975	7	10	
72	Ganguli et al 1975	8	10	
73	Ganguli et al 1975	9	10	
74	Ganguli et al 1975	10	10	
75	Ganguli et al 1975	11	16	50
76	Ganguli et al 1975	12	16	
77	Ganguli et al 1975	13	16	
78	Ganguli et al 1975	14	16	
79	Ganguli et al 1975	15	16	
80	Genin et al 2008	1	10	
81	Genin et al 2008	2	9	
82	Genin et al 2008	3	13	
83	Gjovaag et al 2014	1	12	52,8
84	Gjovaag et al 2014	2	12	86,4
85	Goktepe et al 2010	1	9	25
86	Goktepe et al 2010	2	9	50
87	Goktepe et al 2010	3	9	25
88	Goktepe et al 2010	4	9	50
89	Goktepe et al 2010	5	31	25
90	Goktepe et al 2010	6	31	50
91	Goktepe et al 2010	7	31	25
92	Goktepe et al 2010	8	31	50
93	Hagberg et al 2007	1	41	61,7
94	Hagberg et al 2007	2	22	90
95	Hagberg et al 2010	1	28	65,1
96	Hagberg et al 2010	2	28	67,8
97	Hagberg et al 2010	3	31	84,5
98	Hagberg et al 2010	4	31	87,1
99	Hamamura et al 2008	1	44	

100	Hamamura et al 2008	2	20	
101	Hoffman et al 1997	1	5	49,2
102	Hoffman et al 1997	2	5	18
103	Hoffman et al 1997	3	5	36,6
104	Hoffman et al 1997	4	5	54,6
105	Hoffman et al 1997	5	5	62,4
106	Hoffman et al 1997	6	5	18
107	Hoffman et al 1997	7	5	36,6
108	Hoffman et al 1997	8	5	54,6
109	Houdijk et al 2009	1	11	81,7
110	Houdijk et al 2009	2	11	78
111	Houdijk et al 2009	3	11	91,7
112	Houdijk et al 2009	4	11	78
113	Huang et al 1976	1	25	
114	Huang et al 1976	2	6	
115	Huang et al 1976	3	6	
116	Huang et al 1976	4	4	
117	Isakov et al 1985	1	14	
118	Isakov et al 1985	2	3	
119	Jaegers et al 1993	1	11	
120	Jaegers et al 1993	2	6	
121	James et al 1973	1	37	25
122	James et al 1973	2	37	45
123	James et al 1973	3	37	65
124	James et al 1973	4	37	25
125	James et al 1973	5	37	45
126	James et al 1973	6	37	65
127	James et al 1973	7	26	25
128	James et al 1973	8	26	45
129	James et al 1973	9	26	65
130	James et al 1973	10	26	25
131	James et al 1973	11	26	45
132	James et al 1973	12	26	65
133	Kark et al 2011	1	6	
134	Kark et al 2011	2	10	
135	Kark et al 2011	3	28	
136	Kaufman et al 2008	1	15	27
137	Kaufman et al 2008	2	15	27
138	Kaufman et al 2008	3	15	54
139	Kaufman et al 2008	4	15	54
140	Kaufman et al 2008	5	15	81
141	Kaufman et al 2008	6	15	81
142	Mohanty et al 2012	1	30	63,3
143	Mohanty et al 2012	2	30	63
144	Nowroozi et al 1983	1	8	
145	Nowroozi et al 1983	2	8	
146	Nowroozi et al 1983	3	8	
147	Nowroozi et al 1983	4	8	
148	Nowroozi et al 1983	5	10	
149	Nowroozi et al 1983	6	10	

150	Nowroozi et al 1983	7	10	
151	Nowroozi et al 1983	8	10	
152	Nowroozi et al 1983	9	11	
153	Pagliarulo et al 1979	1	15	71
154	Pagliarulo et al 1979	2	15	71
155	Paysant et al 2006	3	10	77,5
156	Paysant et al 2006	2	10	90
157	Paysant et al 2006	1	10	91,4
158	Paysant et al 2006	5	10	88,1
159	Paysant et al 2006	6	10	74,7
160	Paysant et al 2006	4	10	89,3
161	Pinzur et al 1992	1	25	
162	Pinzur et al 1992	2	5	
163	Rowe et al 2014	1	17	48,3
164	Rowe et al 2014	3	17	68,3
165	Rowe et al 2014	2	17	75
166	Schmalz et al 2002	1	8	67
167	Schmalz et al 2002	2	8	67
168	Schmalz et al 2002	3	8	67
169	Schmalz et al 2002	4	8	67
170	Schmalz et al 2002	5	8	67
171	Schmalz et al 2002	6	8	80
172	Schmalz et al 2002	7	8	80
173	Schmalz et al 2002	8	8	80
174	Schmalz et al 2002	9	8	80
175	Schmalz et al 2002	10	8	80
176	Schmalz et al 2002	11	6	57
177	Schmalz et al 2002	12	6	57
178	Schmalz et al 2002	13	6	48
179	Schmalz et al 2002	14	6	48
180	Schmalz et al 2002	15	6	70
181	Schmalz et al 2002	16	6	70
182	Schnall et al 2012	1	12	80,4
183	Schnall et al 2012	2	12	91,2
184	Schnall et al 2012	3	12	80,4
185	Schnall et al 2012	4	12	91,2
186	Seymour et al 2007	1	10	49
187	Seymour et al 2007	2	10	70
188	Seymour et al 2007	3	10	49
189	Seymour et al 2007	4	10	70
190	Sokhangoei et al 2013	1	24	33,3
191	Sokhangoei et al 2013	2	24	50
192	Sokhangoei et al 2013	3	24	66,67
193	Sokhangoei et al 2013	4	24	33,3
194	Sokhangoei et al 2013	5	24	50
195	Sokhangoei et al 2013	6	24	66,67
196	Tekin et al 2009	1	10	25
197	Tekin et al 2009	2	10	50
198	Tekin et al 2009	3	10	25
199	Tekin et al 2009	4	10	50

200	Tekin et al 2009	5	9	25
201	Tekin et al 2009	6	9	50
202	Tekin et al 2009	7	9	25
203	Tekin et al 2009	8	9	50
204	Torburn et al 1995	1	9	82,3
205	Torburn et al 1995	2	9	82,3
206	Torburn et al 1995	3	9	82,3
207	Torburn et al 1995	4	9	82,3
208	Torburn et al 1995	5	9	82,3
209	Torburn et al 1995	6	7	61,7
210	Torburn et al 1995	7	7	61,7
211	Torburn et al 1995	8	7	61,7
212	Torburn et al 1995	9	7	61,7
213	Torburn et al 1995	10	7	61,7
214	Trabellesi et al 2008	2	8	17,4
215	Trabellesi et al 2008	1	8	39,6
216	Trabellesi et al 2008	4	16	11,4
217	Trabellesi et al 2008	3	16	27
218	Vllasolli et al 2014	1	22	60
219	Vllasolli et al 2014	2	61	75
220	Vllasolli et al 2014	3	6	85
221	Waters et al 1976	1	13	36
222	Waters et al 1976	2	13	48
223	Waters et al 1976	3	13	45
224	Waters et al 1976	4	13	39
225	Waters et al 1976	5	15	54
226	Waters et al 1976	6	15	39
227	Waters et al 1976	7	15	52
228	Waters et al 1976	8	15	65
229	Waters et al 1976	9	14	71
230	Waters et al 1976	10	14	71
231	Waters et al 1976	11	50	
232	Wezenberg et al 2013	1	26	41,7
233	Wezenberg et al 2013	2	10	58,3
234	Wezenberg et al 2013	3	21	75
235	Wright et al 2008	1	10	58,3
236	Wright et al 2008	2		81,6
237	Andrysek et al, 2011	1	14	
238	Andrysek et al, 2016	1	10	64,2
239	Andrysek et al, 2016	2	10	80,4
240	Andrysek et al, 2016	3	10	64,8
241	Andrysek et al, 2016	4	10	77,4
242	Delussu et al, 2016	1	20	40
243	Delussu et al, 2016	2	20	43
244	Esposito et al, 2016	1	6	74,4
245	Esposito et al, 2016	2	6	74,4
246	Esposito et al, 2016	3	6	72,6
247	Esposito et al, 2016	4	6	74,4
248	Esposito et al, 2016	5	6	74,4
249	Esposito et al, 2016	6	6	72,6

250	Guirao et al, 2017	1	10	49,2
251	Guirao et al, 2017	2	10	61,2
252	Lacraz et al, 2016	1	14	77
253	Lacraz et al, 2016	2	14	77
254	Weinert et al, 2016	1	8	
255	Weinert et al, 2016	2	9	
256	Weinert et al, 2016	3	10	
257	Weinert et al, 2016	4	10	
258	Starholm et al, 2015	1	8	73,2
259	Starholm et al, 2015	2	8	73,2
260	Starholm et al, 2015	3	8	54
261	Starholm et al, 2015	4	8	53,4
262	Starholm et al, 2015	5	8	91,2
263	Starholm et al, 2015	6	8	91,2
264	Starholm et al, 2015	7	8	79,8
265	Starholm et al, 2015	8	8	79,8
266	Ladlow et al, 2017	1	10	
267	Ladlow et al, 2017	2	10	
268	Ladlow et al, 2017	3	10	
269	Jarvis et al, 2017	1	10	81,2
270	Jarvis et al, 2017	2	10	73,2
271	Jarvis et al, 2017	3	10	67,2
272	Jarvis et al, 2017	4	10	77,4
273	Gjovaag et al, 2017	1	8	73,2
274	Gjovaag et al, 2017	2	8	
275	Gjovaag et al, 2017	3	8	
276	Gjovaag et al, 2017	4	8	
277	Gjovaag et al, 2017	5	8	
278	Gjovaag et al, 2017	6	8	91,3
279	Gjovaag et al, 2017	7	8	
280	Gjovaag et al, 2017	8	8	
281	Gjovaag et al, 2017	9	8	
282	Gjovaag et al, 2017	10	8	
283	Esposito et al, 2017	1	14	43,2
284	Esposito et al, 2017	2	14	58,2
285	Esposito et al, 2017	3	14	72,6
286	Esposito et al, 2017	4	14	87
287	Esposito et al, 2017	5	14	73,8
288	Esposito et al, 2017	6	14	43,8
289	Esposito et al, 2017	7	14	58,2
290	Esposito et al, 2017	8	14	72,6
291	Esposito et al, 2017	9	14	87
292	Esposito et al, 2017	10	14	80,4
293	Gardinier et al, 2017	1	10	76,8
294	Gardinier et al, 2017	2	10	78,6
295	Gardinier et al, 2017	3	10	76,8
296	Mutlu et al, 2017	1	13	

mean energy	SD energy
17,3	2,7
17,3	5
8,7	1,9
10,4	2,5
13,3	3,1
17,3	3,2
11,6	2,6
15,6	4,3
20,1	3,6
26,9	5,2
12,4	5,7
16,3	4
21	4,3
28,1	5,4
9,5	1,1
10,9	0,9
12,7	1,1
15,5	1,6
19,1	2,3
9,6	1,02
10,9	1
12,8	1,1
15,5	1,3
18,9	1,8
10,4	1,3
15,1	1,9
11,7	2,7
19	5,5
8,5	1,1
11,1	1,9
12,9	
10,9	

12,2	1,6
13,4	1
7,05	1,68
9,34	2,43
7,66	1,78
10,89	2,44
7,73	2,11
10,08	2,21
8,38	1,95
11,21	1,93

	5
	0,64
	0,88
	1,25
11,11	1,66
9,32	2,05
14,47	0,9
10,73	1,36
11,45	3,47
8,81	3,66

13,67	4,88
10,45	2,8
9,76	1,75
15,5	2,8
22,3	4
15,63	0,14
14,05	0,15
13,43	0,21
15,06	0,23
18,29	0,2
14,55	0,19

13,5	0,9
13,3	0,8
13,6	0,7
13,5	0,9
13,6	1,2
16,1	1,4
15,5	1,2
15,7	1,2
15,6	1,3
15,6	1,2
15,1	1,1
14,2	1,2
12,9	0,9
12,1	1,1
16,8	1,4
16,2	2,1
22,2	
26,4	
20,4	
23,8	
12,6	1
13,5	2
16	2
17,2	2

7,48	1,25
9,45	2,13
8,32	1,79
10,3	2,44

7,58	1,22
9,47	2,13
8,32	1,79
10,95	1,4
18,4	3
18	3,6
17,18	3,6
17,08	2,7
17,79	3,5
13,4	2,8
13,61	1,7
13,66	2,7
13,1	2,2
12,41	2,3
12,3	2,5
13,5	2,4
13	3,5
13,2	3,1
12,6	2,9
15	2,9
11,7	1,6
14,6	1,5
11,5	1,5
12,8	4,3
12,9	3,4
15,9	5,4
15,5	2,9
22,4	4,3
13,5	2,2
12,2	2,5
13,8	2,1
14	4
13	4
13,4	0,9
11,3	0,9
12,2	1,2
23,1	2,5
21,6	0,9
20,9	2,2

15,8	3,5
15,6	2,8
12,4	1,5
12,4	2,1
14,6	1,9
15,5	2,6
13,2	4
13,4	4,4
12,3	
13,3	
16,2	
11,3	
15,9	
14,1	
13,7	2,4
15,8	2
18,7	2,1
22,7	2
19,2	3,2
9,6	1
10,9	0,9
12,7	1,2
15,5	1,3
14,4	2
14,5	1,9
14,3	1,7
13,3	0,8
